# Clinical Features, Genetics and Treatment in a Russian Cohort of Young Patients with Xeroderma Pigmentosum: A Single Center Experience

**DOI:** 10.3390/ijms27219889

**Published:** 2026-11-04

**Authors:** Tatiana Belysheva, Tatiana Nasedkina, Ekaterina Zelenova, Vera Semenova, Peter Sparber, Elena Sharapova, Denis Sofronov, Marina Dorofeeva, Olga Bozhko, Yana Vishnevskaya, Irina Kletskaya, Svetlana Mikhaylova, Timur Valiev, Natalia Petenko, Kristina Orlova, Igor Samoilenko, Svetlana Varfolomeeva

**Affiliations:** 1 N.N. Blokhin National Medical Research Center of Oncology, 115478 Moscow, Russia; klinderma@bk.ru (T.B.); zelenovayeye@gmail.com (E.Z.); sharapovae.v@yandex.ru (E.S.); mdsofronov@gmail.com (D.S.); yana_vishn@list.ru (Y.V.); astra-sn@mail.ru (S.M.); timurvaliev@mail.ru (T.V.); n.petenko@gmail.com (N.P.); krisman03@gmail.com (K.O.); i.samoylenko@ronc.ru (I.S.); s.varfolomeeva@ronc.ru (S.V.); 2 Engelhardt Institute of Molecular Biology of the Russian Academy of Sciences, 119991 Moscow, Russia; sulpiridum@yandex.ru; 3 Dmitry Rogachev National Medical Research Center of Pediatric Hematology, Oncology and Immunology, 117198 Moscow, Russia; 4 Research Center for Medical Genetics, 115522 Moscow, Russia; psparber93@gmail.com; 5 Veltischev Research and Clinical Institute for Pediatrics and Pediatric Surgery, Pirogov Russian National Research Medical University, 125412 Moscow, Russia; mdorofeeva@inbox.ru (M.D.); bozhko_olga@mail.ru (O.B.); 6 Russian Children’s Clinical Hospital of Pirogov Russian National Research Medical University, 119571 Moscow, Russia; ikletskaya@gmail.com; 7 Department of Oncology, Sechenov First Moscow State Medical University, 119048 Moscow, Russia; 8 National Medical Research Radiological Center of the Ministry of Health of the Russian Federation, 125284 Moscow, Russia; 9 Department of Oncology, Radiation Therapy and Radiology named after Professor N.E. Yakhontov, Privolzhsky Research Medical University, 603005 Nizhny Novgorod, Russia

**Keywords:** xeroderma pigmentosum, basal cell carcinoma, squamous cell carcinoma, melanoma, vismodegib, sonidegib, pembrolizumab, *XPC*, *XPA*, *ERCC6*, *DDB2*

## Abstract

Xeroderma pigmentosum (XP) and Cockayne syndrome (CS) are rare autosomal recessive DNA repair disorders. XP is characterized by extreme sensitivity to UV radiation and a very high risk of skin cancer. To date, little is known about the clinical characteristics and genetic basis of these diseases in Russia. Here, we present a detailed study of 14 patients (aged 6–30 years) from 11 unrelated families of diverse ethnic backgrounds. Using a multigene panel or whole-genome sequencing, we identified patients with homozygous or compound heterozygous variants in *XPC* (*n* = 8), *XPA* (*n* = 4), *DDB2* (*n* = 1), and *ERCC6* (*n* = 1). Two XP and one CS cases were presented in one family. A total of 110 relatives were screened to determine carrier status. Skin cancer was revealed in 100% of the 13 XP patients; the median age at first manifestation was 13 years (range 5–23). Basal cell carcinoma (BCC), squamous cell carcinoma (SCC), and melanoma developed in 10, 7, and 3 patients, respectively. All three cancer types occurred in two patients. The patients underwent surgical treatment; additionally, two patients with BCC received targeted therapy (vismodegib, sonidegib). Anti-PD-1 immunotherapy was administered successfully in three patients with multiple or advanced skin tumors.

## 1. Introduction

The nucleotide excision repair (NER) pathway can be divided into the slow global genome nucleotide excision repair (GG-NER) and the rapid transcription-coupled nucleotide excision repair (TC-NER) subpathways. The predominant difference between GG-NER and TC-NER is the damage recognition step. While TC-NER is initiated by blocked RNA polymerases at photolesions on the transcribed DNA strand, the initial damage recognition in GG-NER is performed by the XPC complex (XPC, RAD23B and centrin-2), which continuously scans the entire genome and detects structures that disrupt Watson–Crick base pairing [1].

Xeroderma pigmentosum (XP) is a rare genodermatosis with autosomal recessive inheritance and complete penetrance [1,2]. The disease is caused by homozygous or compound heterozygous pathogenic variants in the genes of nine complementation groups: XP-A (*XPA*), XP-B (*ERCC3*), XP-C (*XPC*), XP-D (*ERCC2*), XP-E (*DDB2*), XP-F (*ERCC4*), XP-G (*ERCC5*), XP-V (*POLH*), and XP-J (*GTF2H4*), involved in GG-NER [2,3,4].

The incidence of XP in European populations and the USA is 1–2 per 1,000,000 [5,6]. It is more common in some isolated countries such as Japan (1–5 per 100,000) [7] or Pakistan [8]. One of the highest incidences of XP (>1/50,000) occurs in Tunisia, Algeria, and Morocco (hereafter referred to as North Africa) and is associated with a high allelic frequency of a founder *XPC* mutation in populations with traditions of consanguinity [9,10].

The classic manifestation of XP is ultraviolet-induced progressive skin lesions with the development of lentigines, poikiloderma, xerosis, atrophy and cheilitis, as well as ocular involvement, including dryness, lacrimation, photophobia, and ophthalmic sclerosis [2,11,12]. Skin cancer develops in the majority of patients, with basal cell carcinoma (BCC) being the most common type. Squamous cell carcinoma (SCC) and melanoma are also frequently observed, and a combination of several types of tumors is possible in one patient, including basosquamous carcinoma (BSC) [13,14].

According to previously described genotype–phenotype correlations, patients with pathogenic variants in the *XPA*, *ERCC2*, and *ERCC5* genes often exhibit neurological disorders (progressive neurodegeneration, developmental delay, ataxia, sensorineural hearing loss, and brain atrophy) [15,16,17]. Patients with pathogenic variants in the *XPC* gene are at increased risk of developing extracutaneous malignancies (hematologic malignancies, central nervous system, gynecological, and thyroid tumors) [18,19,20]. Pathogenic variants in the *ERCC4*, *POLH*, and *DDB2* genes are associated with a milder disease course and a relatively late onset of skin tumors [15,21].

The TC-NER pathway repairs DNA damage specifically in actively transcribed regions of the genome [22]. Homozygous or compound heterozygous pathogenic variants in the key TC-NER genes, *CSA* (*ERCC8*) or *CSB* (*ERCC6*), cause Cockayne syndrome, which is characterized by developmental delay and signs of accelerated aging [23]. Since excision repair proteins such as XPB/ERCC3, XPD/ERCC2, XPG/ERCC5, and XPF/ERCC4 are involved in both GG-NER and TC-NER, overlapping phenotypic manifestations or combined phenotypes with features of both XP and Cockayne syndrome are possible [24,25]. Molecular analysis of the disease plays an important role in establishing an accurate diagnosis, determining the prognosis, and selecting treatment strategies for patients [15].

For the treatment of patients with XP, surgical removal of skin cancer [26], radiation therapy [27], photodynamic therapy [28], immune checkpoint inhibitor (ICI) treatment [29], targeted therapy with Hedgehog signaling pathway inhibitors [30], and topical agents such as imiquimod [31] and 5-fluorouracil [32] are used. However, due to multiple lesions and poor adherence to preventive measures (complete sun avoidance, regular follow-up, and lifelong symptomatic treatment), life expectancy often does not exceed 40 years.

The clinical and genetic characteristics of patients with XP have been described in detail in many populations from Asia, Europe, North Africa, North and South America [5,6,7,8,9,10]. However, in Russia, only isolated cases of XP have been reported to date, usually without molecular confirmation [33,34,35]. Therefore, the prevalence of XP in the Russian Federation, as well as the genetic basis of the disease, remains virtually unstudied. This article presents the clinical and genetic characteristics, management, and treatment experience (surgical treatment, immunotherapy, and targeted therapy) of 13 patients with XP from 11 families representing various ethnic groups in the Russian Federation. We also describe a unique case in one consanguineous family with pathogenic variants in the *XPC* and *ERCC6* genes, resulting in two children with XP and one child with Cockayne syndrome. Given the multinational composition of the population of Russia and possible differences in the mutational spectrum compared to other countries, the presented data are of scientific value and complement the global cohort of patients with XP. These results can also be used to clarify genotype–phenotype correlations in XP.

## 2. Results

### 2.1. Study Population

**Figure 1:**
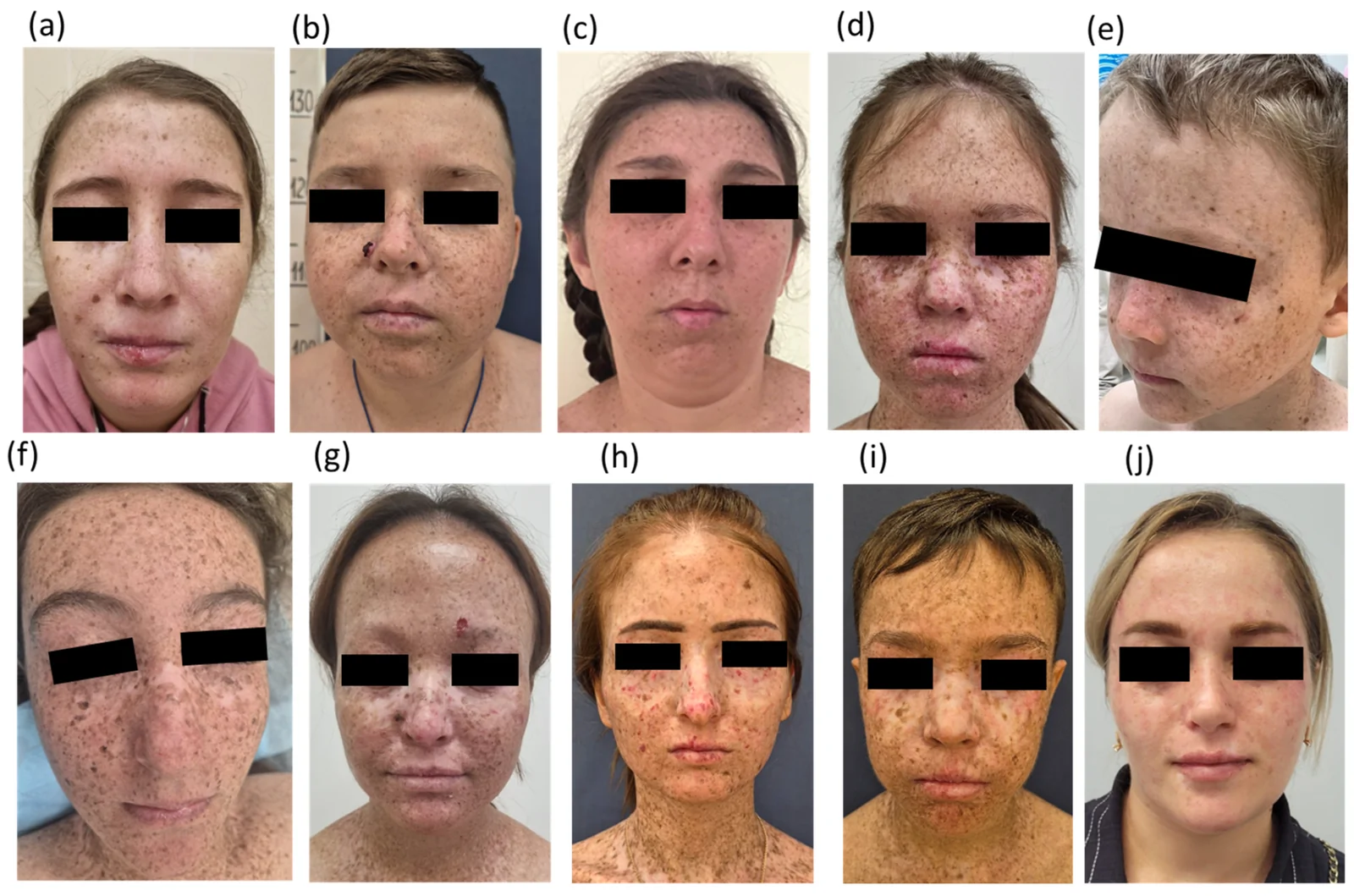
Phenotypes of the patients involved in the study. XP patients (**a**–**j**). Complementation group XP-A: (**a**)—ID1 (at age 11 years), (**b**)—ID2 (10 years), (**c**)—ID3 (14 years). Complementation group XP-C: (**d**)—ID4 (14 years), (**e**)—ID5 (5 years), (**f**)—ID6 (14 years), (**g**)—ID7 (20 years), (**h**)—ID8 (18 years), (**i**)—ID9 (12 years). Complementation group XP-E: (**j**)—ID10 (30 years).

In total, 14 patients were enrolled (13 XP patients and one patient with CS): seven males and seven females, including children, adolescents, and young adults, with a median age of 16.5 years (range 6–30) at present. The patients belonged to 11 unrelated families and were of various ethnic origins (Table 1 and Table S1, Figure 1). 

Consanguinity was reported in 10/14 (71%) patients. The first symptoms in XP patients were photodermatitis, lentigo, and photophobia, with a median age at presentation of 12 months (range 1–24). Patient ID3 was reported earlier at age 8, and only skin lesions (chronic dermatitis with melanosis) were described [33]. More detailed examination at age 14 revealed coarse facial features, short neck, micrognathia, scoliosis, and pronounced neurologic deficit (Figure 1c). Cockayne syndrome manifested with nystagmus and cataract during the first 6 months of life, followed by dry skin and developmental delay at the age of 3 years.

**Figure 2:**
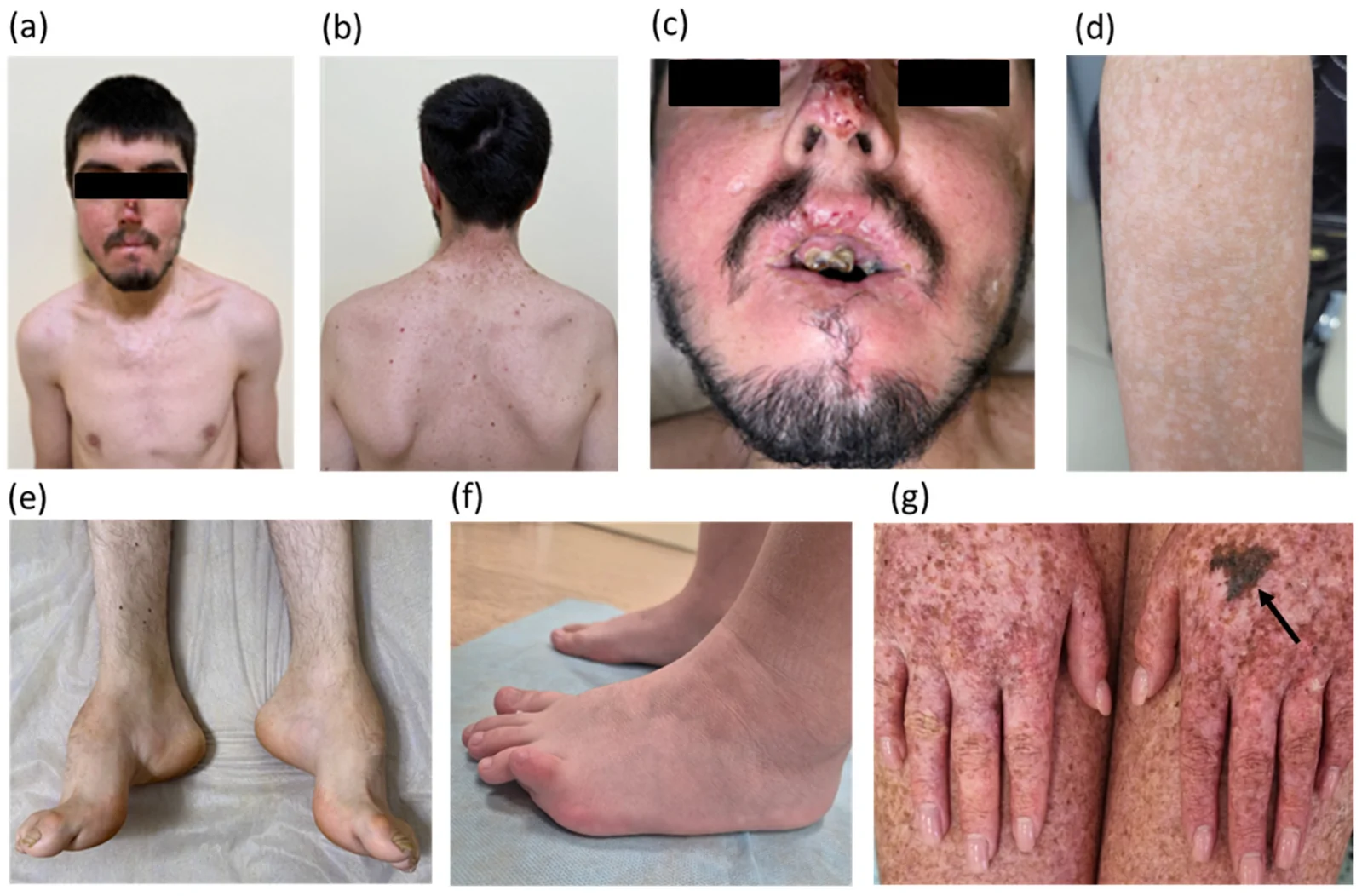
Phenotypic characteristics of XP patients. Patient ID11: (**a**,**b**) general appearance (age 27 years); (**c**) facial features: deformation and narrowing of the oral opening, numerous fissures, dryness and atrophic scar-like changes, a scar on the chin resulting from melanoma removal, and a malignant tumor of the nose. Foot deformities: (**e**) patient ID11 and (**f**) patient ID2. Skin lesions: (**d**) dyschromia, multiple areas of hypopigmentation, and skin atrophy on the hands (patient ID10); (**g**) pronounced lentiginosis on sun-exposed and lentigo maligna melanoma, stage TisN0M0 (arrow) in patient ID8.

The median follow-up time was 3 years (range 1–6). At the first visit to the Blokhin National Medical Research Center of Oncology, skin lesions in XP patients included classic manifestations such as multiple ephelides, lentigo, poikiloderma, xerosis cutis, atrophy, and cheilitis (Figure 1 and Figure 2); in addition, some patients had SCC as the first cancer (Figure 1a,c–e,h,i), BCC (Figure 1b,d,f,g,j), and melanoma (Figure 1h).

Other characteristic features of XP patients are presented in Figure 2. Patient ID11 had severe underweight (height 165 cm and weight 43 kg at the age of 27 years), deformation and narrowing of the oral opening, melanoma of the chin (surgically removed), malignancy of the nose, BCC in the area of the left superciliary arch, and foot deformities since the age of 10 (Figure 2a–c,e). The first superficial clinical description of this patient at age 17 was presented earlier, but no thorough examination and genetic analysis were performed [34]. Valgus deformity of the feet was also presented in patient ID2 (Figure 2f). Dyschromia, multiple areas of hypopigmentation and skin atrophy were noted on exposed areas of the skin (patient ID10) (Figure 2d). The role of UV radiation in the development of skin pigmentation is clearly visible when comparing the pronounced picture of lentiginosis of the hands (Figure 2g) (patient ID8) and the absence of pigmentation on closed areas of the skin, for example, on the chest and the back (patient ID11) (Figure 2a,b).

In XP patients, the most common cancer was BCC (10/13; 77%), followed by SCC (7/13; 54%); in one case, BCC and SCC developed synchronously. Collision tumor (BCC and SCC) and basosquamous carcinoma (BSC) were found in one patient (8%). Cutaneous melanoma developed in 23% (3/13) of patients, with the earliest onset at the age of 13 years. In XP patients, neurological impairment or psychiatric disorders were found in 6/13 (46%), and ophthalmological disorders occurred in 7/13 (54%) of cases.

Patient ID14 with Cockayne syndrome had short stature (116 cm) and low weight (24 kg), microcephaly (head circumference 48 cm), facial dysmorphism (narrow face, thin nose, sunken eyes, and protruding ears), a history of cataract surgery, pigmentary degeneration of the retina, xerosis cutis, areas of hyperpigmentation on the face and neck, dry hair, shortening of the metacarpal bones, and joint stiffness. There was significant underdevelopment of speech, intellectual disability, hearing loss, and a stiff, intermittently unsteady gait.

### 2.2. Molecular Genetic Findings

In the first stage, all patients underwent genetic testing by targeted NGS using a multigene panel that included the XP (*DDB2*, *ERCC2*, *ERCC3*, *ERCC4*, *ERCC5*, *POLH*, *XPA*, and *XPC*) and CS (*ERCC6* and *ERCC8*) genes (Table S2). Targeted sequencing identified a total of 12 different candidate variants of the disease: five *XPA* variants, four in the *XPC* gene, two in the *ERCC6* gene, and one variant in the *DDB2* gene (Table 2 and Table S3).

In family 11, multigene panel sequencing revealed only two heterozygous variants in the *ERCC6* gene in patient ID14 with clinical features of Cockayne syndrome, whereas no genetic changes in the targeted genes were found in patients ID12 and ID13, who had a clinical diagnosis of XP. Subsequent clinical exome sequencing also yielded a negative result.

**Figure 3:**
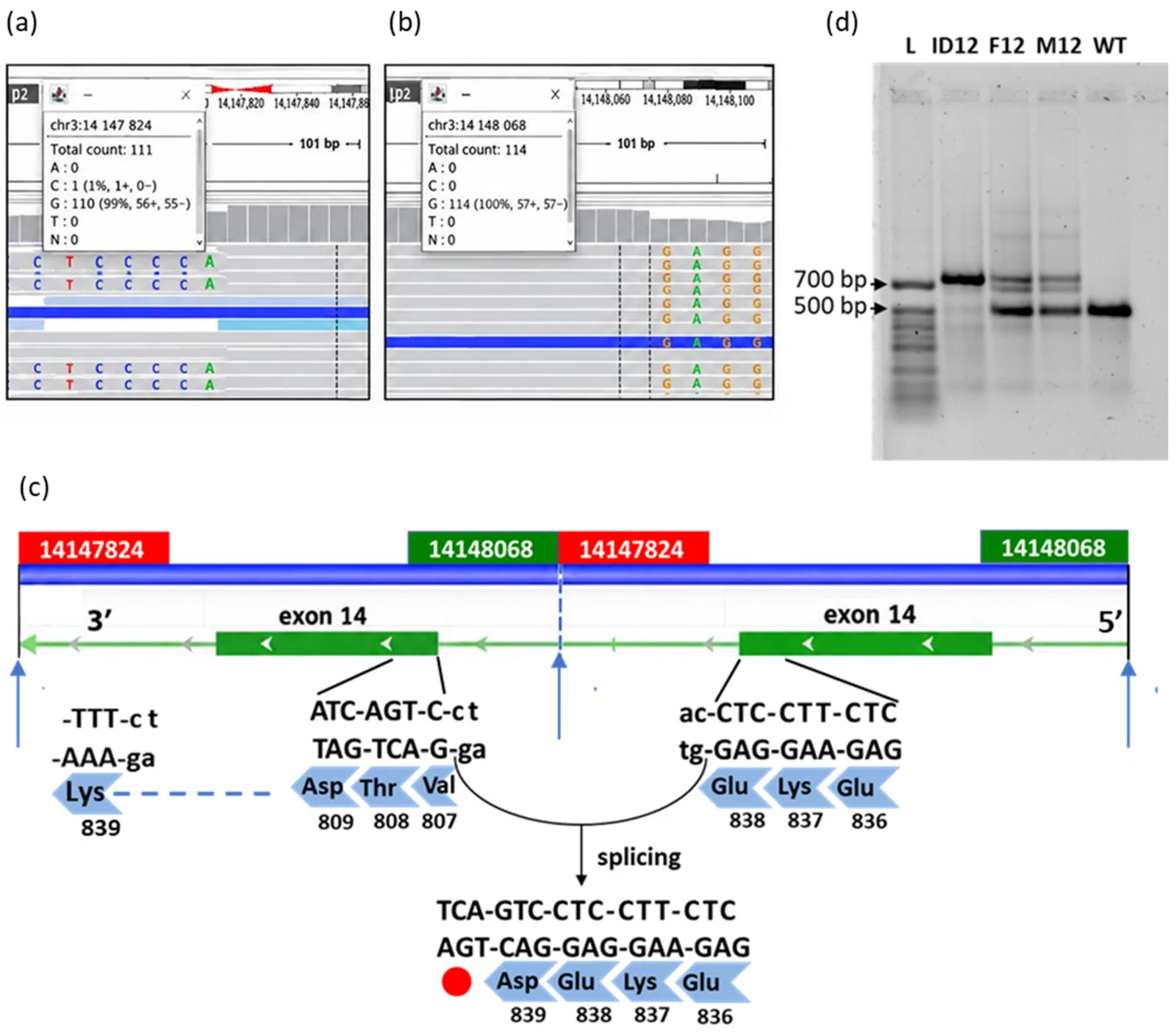
Homozygous duplication in *XPC* identified in two relatives with XP from family 11. Visualization of sequencing results using Integrative Genomics Viewer (IGV): the start (**a**) and end (**b**) positions of the duplicated region. (**c**)—Intragenic tandem duplication of exon 14 leading to a translational frameshift and a premature stop codon. (**d**)—Agarose gel electrophoresis of exon 14 PCR products (patient ID 12). Lanes: L, DNA ladder; ID12, patient; F12, his father; M12, his mother; WT, wild-type control. The duplication yields a 692-bp PCR product from genomic DNA, whereas the wild-type product is 447 bp. The parents are heterozygous and carry both the 447-bp and 692-bp products. A similar pattern was observed for patient ID13 and his parents (Figure S1).

We performed trio whole-genome sequencing (WGS) for patient ID12, and a large genomic duplication in the exon 14 region of the *XPC* gene was detected (Figure 3a–c). Bioinformatic analysis identified the coding sequence c.2421_2514dup, which was an intragenic tandem duplication of 94 nucleotides within exon 14. This alteration leads to a frameshift starting at codon 839 and a premature stop codon two amino acids downstream (p.Lys839Aspfs*2). The presence of a homozygous 245 bp duplication in the genome sequence was confirmed by PCR and electrophoresis in patients ID12 and ID13, while the parents of these patients were heterozygous carriers (Figure 3d and Figure S1).

**Figure 4:**
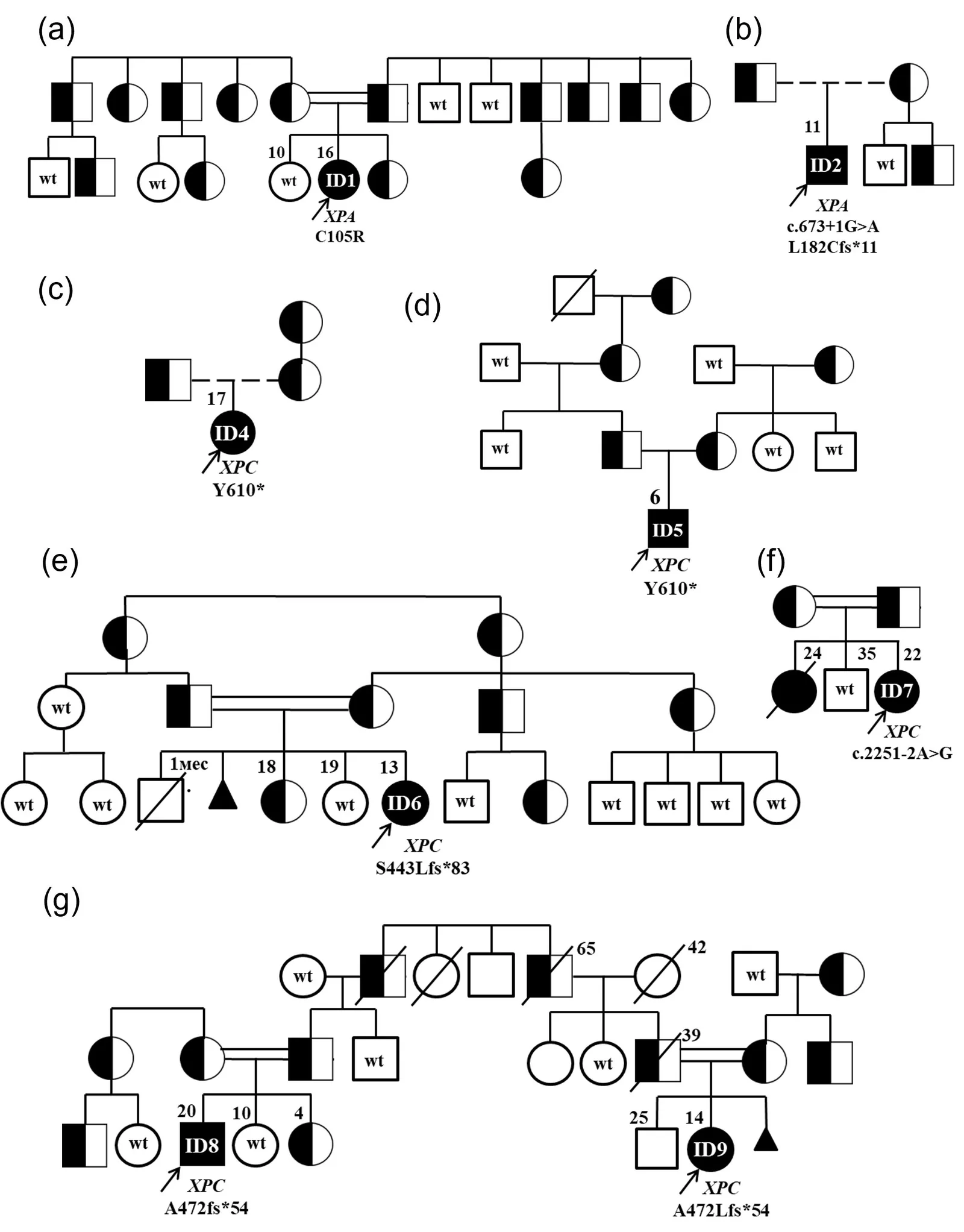
Examples of pedigrees based on segregation analysis. (**a**)—family 1 (ID1), (**b**)—family 2 (ID2), (**c**)—family 4 (ID4), (**d**)—family 5 (ID5), (**e**)—family 6 (ID6), (**f**)—family 7 (ID7), (**g**)—family 8 (ID8 and ID9).

**Figure 5:**
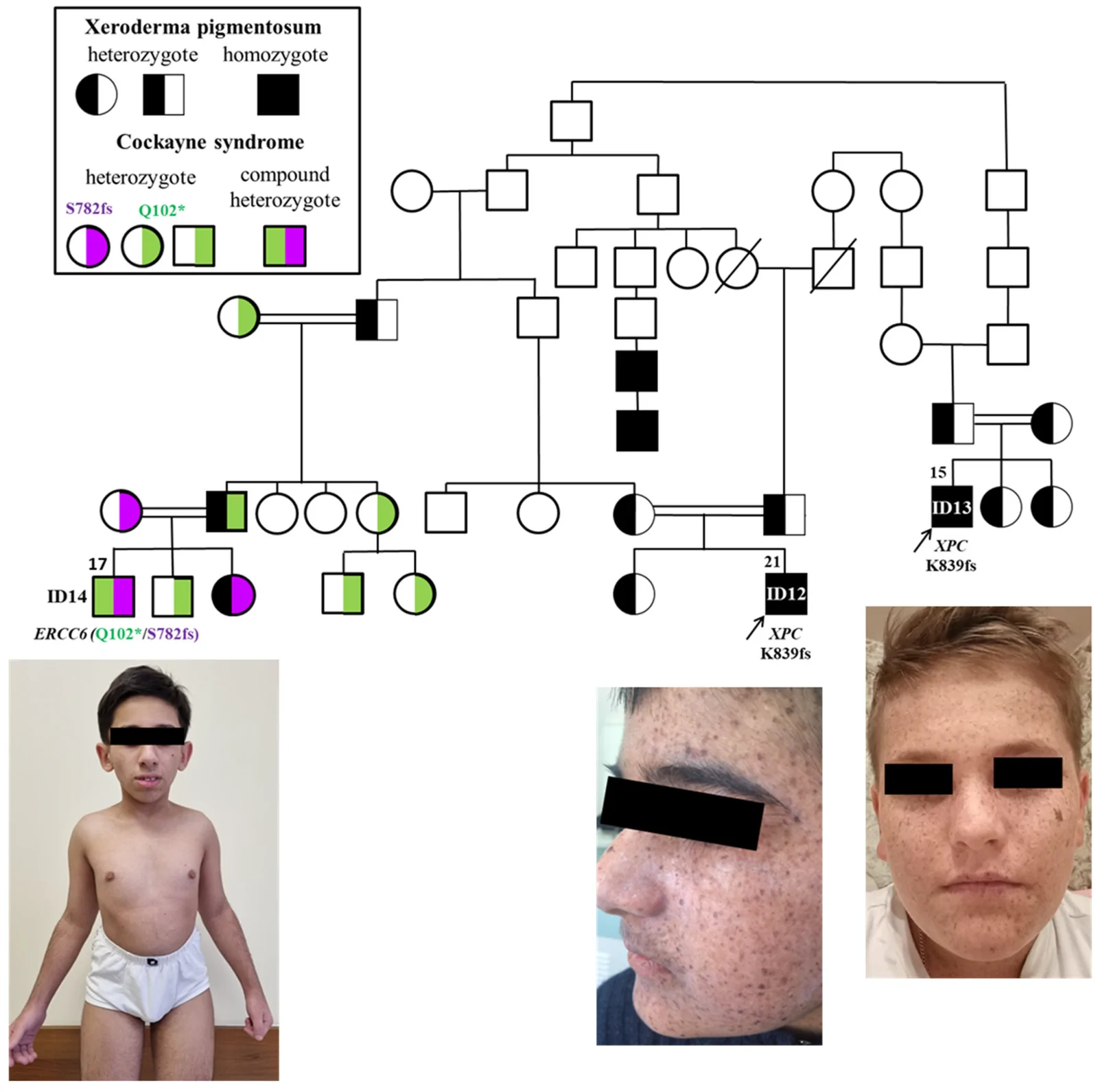
Pedigree of family 11: patients ID12 (at age 17 years) and ID13 (14 years) with XP and their relative patient ID14 (13 years) with Cockayne syndrome.

Based on the results of segregation analysis in relatives, carrier status was identified in the majority of families (Table S3, Figure 4 and Figure 5).

The same pathogenic variant p.Tyr610* in the *XPC* gene was detected in two unrelated patients (ID4 and ID5) living in the same geographical region (Chuvashia). There was no consanguinity in their families, and the parents of these patients were also carriers of this variant. Theoretically, this may represent a founder effect, which requires confirmation through testing of a larger sample of patients from this region, including haplotype reconstruction.

### 2.3. Neurological Conditions in XP Patients

The majority of patients underwent a comprehensive neurologic examination. Neuropsychiatric or neurological problems were identified in six cases; four of them were associated with *XPA* and two with *XPC* variants.

**Figure 6:**
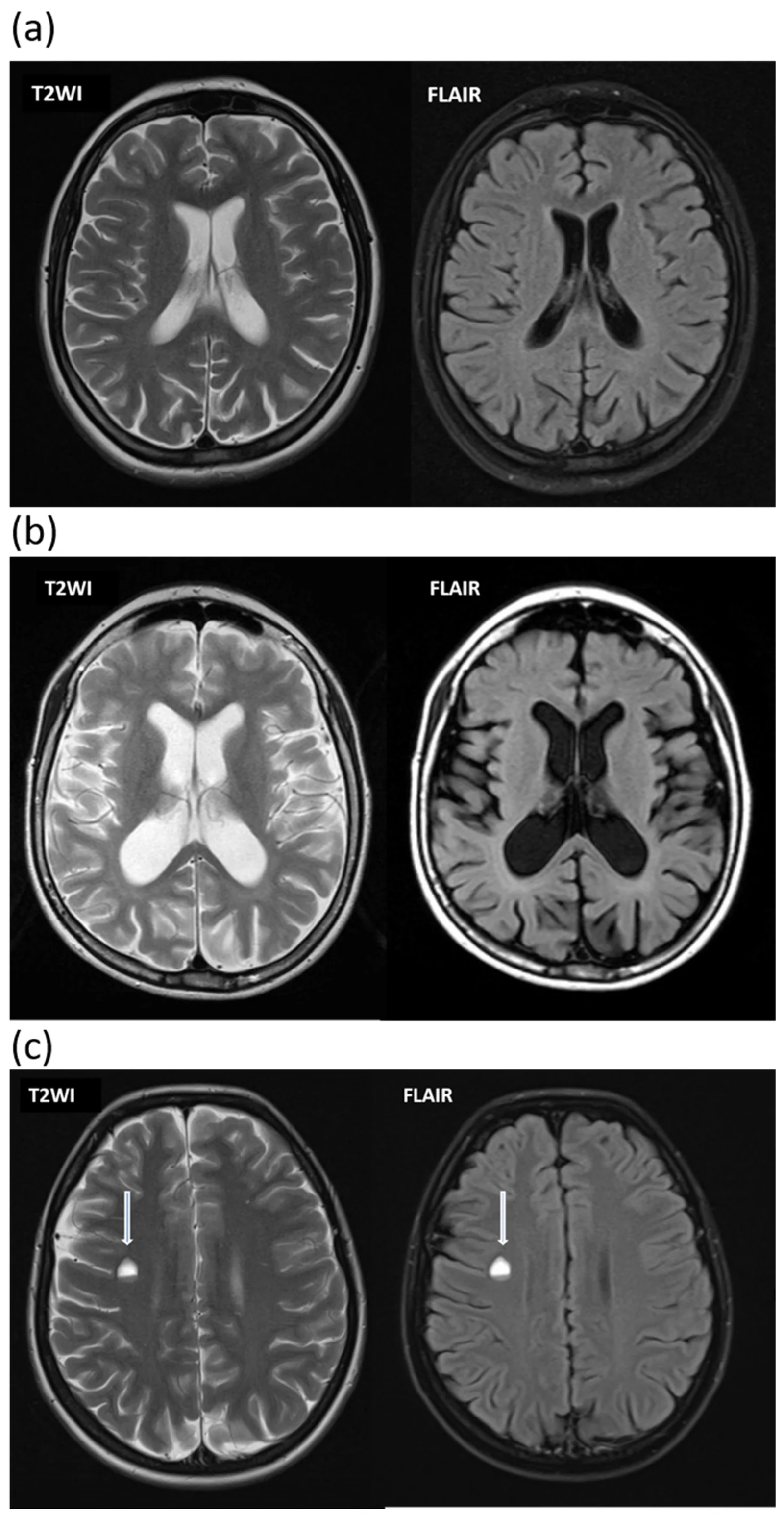
Axial brain MRI scans in T2-weighted imaging (T2WI) (**left**) and fluid-attenuated inversion recovery (FLAIR) (**right**) sequences. (**a**) Patient ID1: mild dilatation of the external and internal cerebrospinal fluid (CSF) spaces. (**b**) Patient ID2: non-tension ventriculomegaly and dilatation of the external CSF spaces. (**c**) Patient ID7: a cyst with hemorrhagic contents and a thin, smooth capsule in the middle third of the right centrum semiovale (arrow).

Patient ID1, who carried a homozygous *XPA* mutation (p.Cys105Arg), exhibited mild intellectual disability, with a Wechsler Intelligence Scale for Children (WISC) Full Scale IQ of 53. Significant static motor impairment was revealed, characterized by an ataxic gait, unsteadiness in the Romberg test, and bilateral intention tremor with impaired coordination. Additionally, mild-to-moderate acrohyperhidrosis was noted, indicating autonomic involvement. Brain MRI revealed no focal abnormalities but demonstrated mild dilatation of the external and internal cerebrospinal fluid (CSF) spaces, consistent with early diffuse cerebral and cerebellar atrophy (Figure 6a).

Patient ID2, carrying compound heterozygous pathogenic variants in the *XPA* gene (c.673+1G>A and p.Leu182Cysfs*11), was diagnosed with moderate intellectual disability (WISC Full Scale IQ of 38). No static motor or coordination impairments were detected. Brain MRI showed moderate dilatation of the CSF spaces and ventriculomegaly (Figure 6b).

Patient ID3, with a homozygous pathogenic variant in *XPA* (p.Arg211*), was diagnosed with mild-to-moderate intellectual disability (WISC Full Scale IQ of 51); in addition, occasional epileptic seizures were recorded. Gait changes characterized by clumsiness and unsteadiness, as well as impaired coordination in both the upper and lower extremities, were also noted. Brain MRI could not be performed because the patient did not fall asleep under sedation and moved, resulting in movement artifacts.

Patient ID5 (*XPC*, p.Tyr610*) was under observation for attention deficit hyperactivity disorder (ADHD). Patient ID7 (*XPC*, c.2251-2A>G) presented with no visible neurological or behavioral symptoms, but a brain MRI revealed a cystic cavernous malformation in the right frontal lobe (Figure 6c).

Patient ID11 (*XPA*, c.172+1G>T) did not undergo a comprehensive examination; however, mild cognitive impairment had been noted since adolescence. Brain MRI was not performed.

### 2.4. Skin Tumors

Multiple diverse skin lesions developed in all patients with XP carrying *XPA*, *XPC*, or *DDB2/XPE* mutations, but not in the patient with CS group B (*ERCC6/CSB*). Benign neoplasms included melanocytic dysplastic nevi (ID1), pyogenic granuloma, superficial atypical melanocytic proliferation of uncertain significance (SAMPUS), and compound nevus (ID8). Skin cancers, namely BCC, SCC, BSC, or melanoma, were diagnosed in all 13 patients with XP.

The XP patients underwent surgical excision of neoplasms as first-line treatment. Among them, two patients (ID7 and ID8) were prescribed targeted therapy with Hedgehog pathway inhibitors; both were given vismodegib, and subsequently, patient ID8 was treated with sonidegib. Three patients received ICI; the same patients, ID7 and ID8, were switched to pembrolizumab after Hedgehog pathway inhibitors. Patient ID11 received nivolumab as an adjuvant treatment after melanoma excision.

#### 2.4.1. Basal Cell Carcinoma in XP Patients

Basal cell carcinoma was the most common type of skin cancer and was diagnosed in 10 of 13 XP patients (77%). The minimum age at onset was 9 years (ID2), and the median age at BCC diagnosis was 12.5 years. A total of 46 tumor lesions were identified in these 10 patients during the follow-up period, with the number of lesions in each patient ranging from 1 to 10.

**Figure 7:**
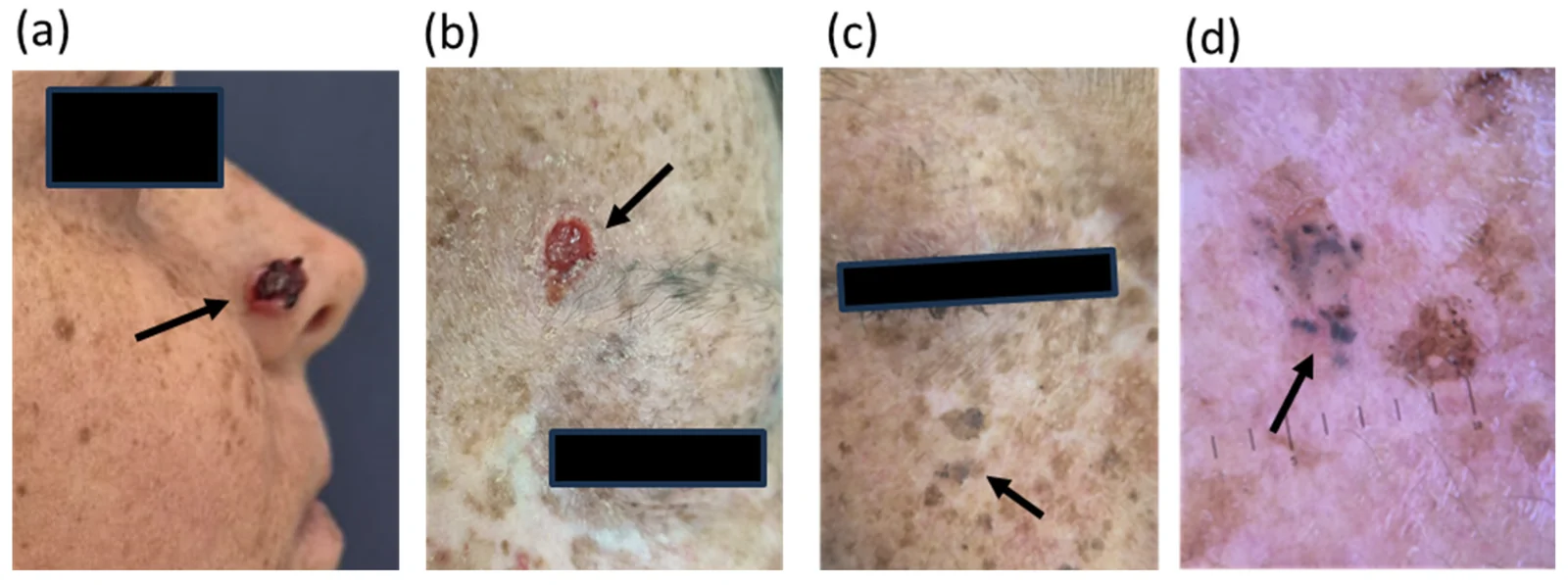
BCC in XP patients (BCC foci are marked by arrows). (**a**)—ID2, (**b**)—ID7, (**c**,**d**)—ID6. (**a**,**b**) show a typical presentation of BCC. Clinical examination (**c**) alone does not permit the diagnosis of BCC, which requires dermatoscopy (**d**).

In most cases (87.5%), BCC was diagnosed at stage T1N0M0, with a predominant location on the face. Patient ID7 had 10 BCC lesions on the face, arms, and trunk (Figure 7b), including two lesions on the face exceeding 2 cm in size that corresponded to stage T2N0M0 (stage II).

**Figure 8:**
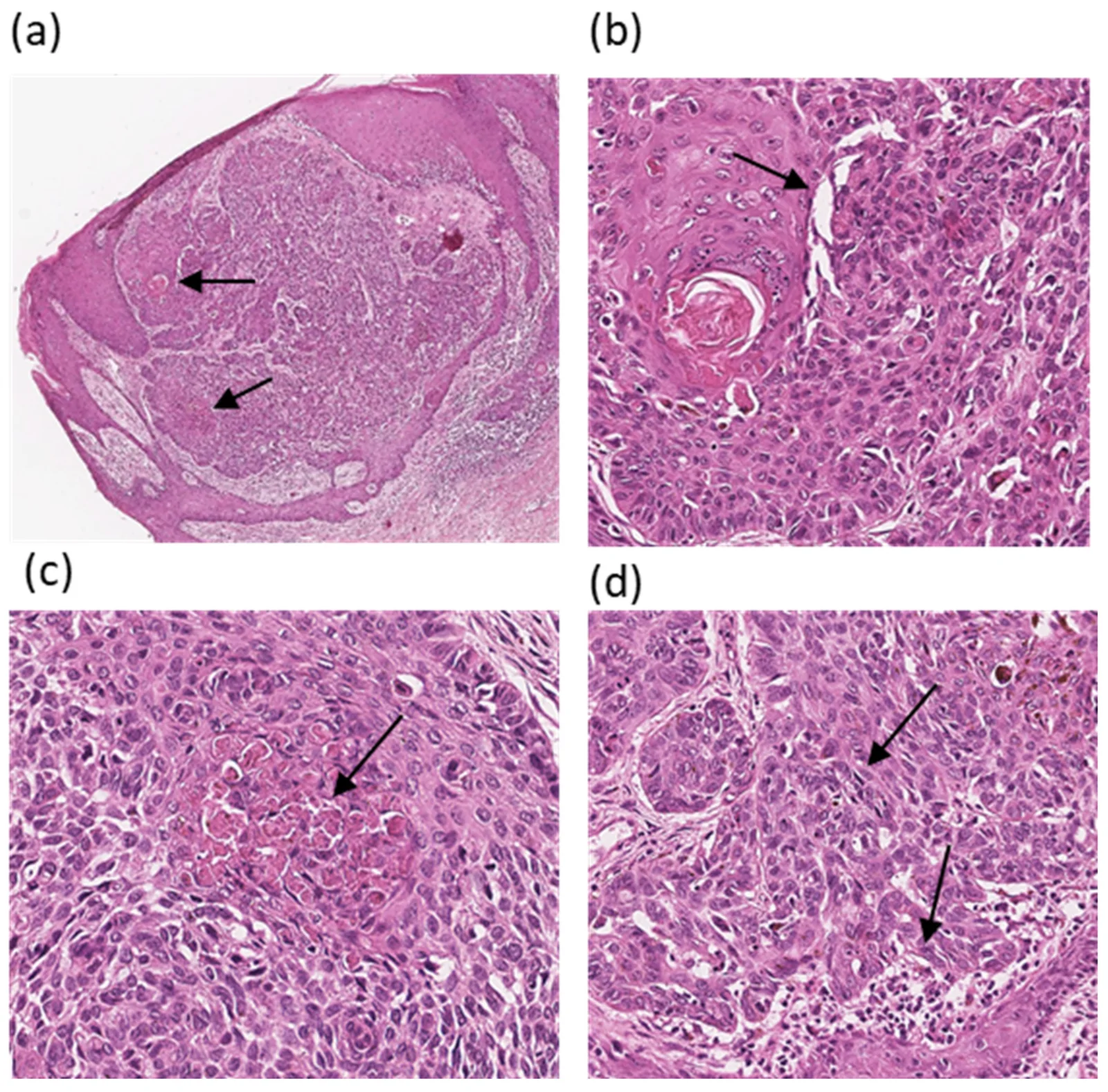
Basosquamous carcinoma developed on the right cheek of patient ID4. (**a**)—Basaloid nodular tumor with foci of squamous differentiation (arrows), H&E, ×100. SCC component: (**b**)—keratin pearl formation (arrow), H&E, ×300. (**c**)—foci of keratinization (arrow) with prominent atypia and multiple mitotic figures, H&E, ×300 (arrow). (**d**)—Areas of basaloid cells with focal melanin deposition (arrows). H&E, ×300. Brightness and contrast were adjusted uniformly across the entire image for visualization purposes only. No selective digital manipulation was applied.

In one case (ID4), BSC was also identified—a rare, highly aggressive form of nonmelanoma skin cancer characterized by the simultaneous presence of BCC and SCC differentiation within a single lesion (Figure 8a). The squamous cell component was characterized by larger, polygonal, atypical cells with abundant eosinophilic cytoplasm. The most important diagnostic criterion is squamous differentiation with the formation of concentric layers (so-called “horny pearls”) and central keratinization (Figure 8b,c). The basal cell component consisted of nests and cords of small anaplastic cells with hyperchromatic nuclei and sparse cytoplasm; peripheral palisading of nuclei was focally preserved at the periphery of the tumor complexes (Figure 8d).

Initially, all patients underwent surgical treatment, including radical excision of facial skin tumors with Mohs micrographic surgery (MMS). Patients ID7 and ID8 initiated treatment with vismodegib (150 mg once daily) for multiple BCC lesions after reaching 18 years of age. They were treated with vismodegib for 40 and 7 months, respectively, and both achieved a complete clinical response. However, patient ID7 subsequently had to discontinue targeted therapy due to BCC recurrence, the development of new BCC lesions, and cutaneous melanoma in situ. Patient ID8 discontinued vismodegib because of clinically significant adverse events (grade 2 diarrhea during the first months of therapy, grade 2–3 myalgia and muscle spasms, grade 1 alopecia, grade 2 headache, grade 2 weakness and fatigue, grade 2 amenorrhea, grade 2 weight loss, and grade 2 decreased appetite), and subsequently began receiving sonidegib (200 mg once daily) as second-line therapy. A complete clinical response was achieved after two months of treatment; however, sonidegib was discontinued after the diagnosis of squamous cell carcinoma (SCC), and the patient was switched to anti-PD-1 immunotherapy.

#### 2.4.2. Squamous Cell Carcinoma in Patients with XP

In this cohort, cutaneous SCC was the second most common malignancy and was diagnosed in 7 of 13 (54%) XP patients. The minimum age at onset for SCC was 5 years (ID5), and the median age was 11 years (range 5–20). The predominant tumor site was the face (100%), with involvement of the shins observed in only one case (ID8). In most patients, all SCC lesions were diagnosed at stage T1N0M0 R0 and were surgically resected.

**Figure 9:**
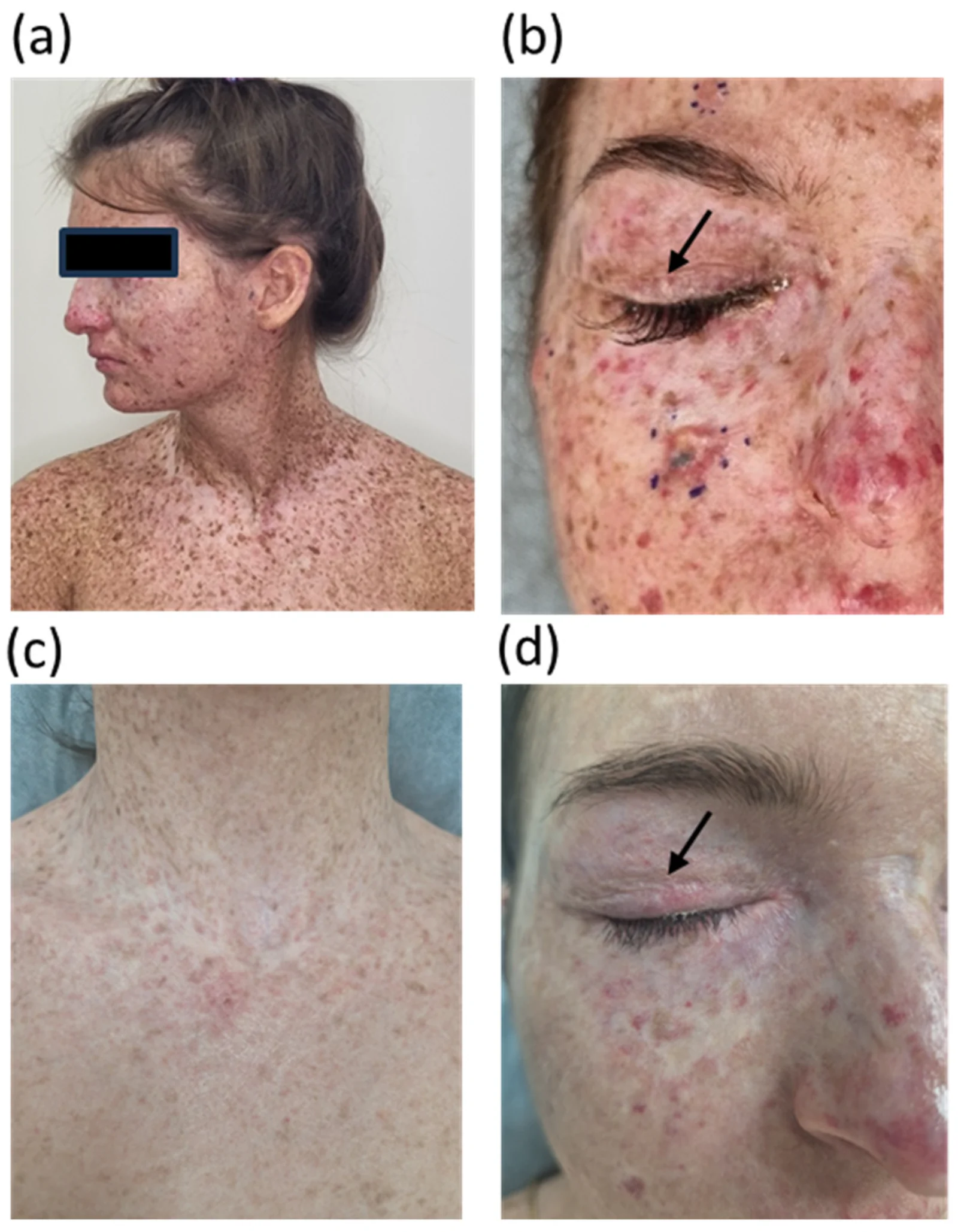
Patient ID8 before (**a**,**b**) and 11 months (**c**,**d**) after ICI (pembrolizumab) therapy. Complete regression of SCC (arrows) and fading of skin lentigines are noticed.

Patient ID8 developed multiple SCCs, including one lesion involving the right upper eyelid. After review by a multidisciplinary team, anti-PD-1 immunotherapy with pembrolizumab was initiated. Treatment has been ongoing for 11 months. A complete response (as assessed clinically) has been achieved. The treatment was well tolerated (the only adverse reaction was grade 2 dermatitis). In addition, a marked reduction in the severity of lentigines, poikiloderma, and xerosis has been observed (Figure 9).

#### 2.4.3. Cutaneous Melanoma in Patients with XP

**Figure 10:**
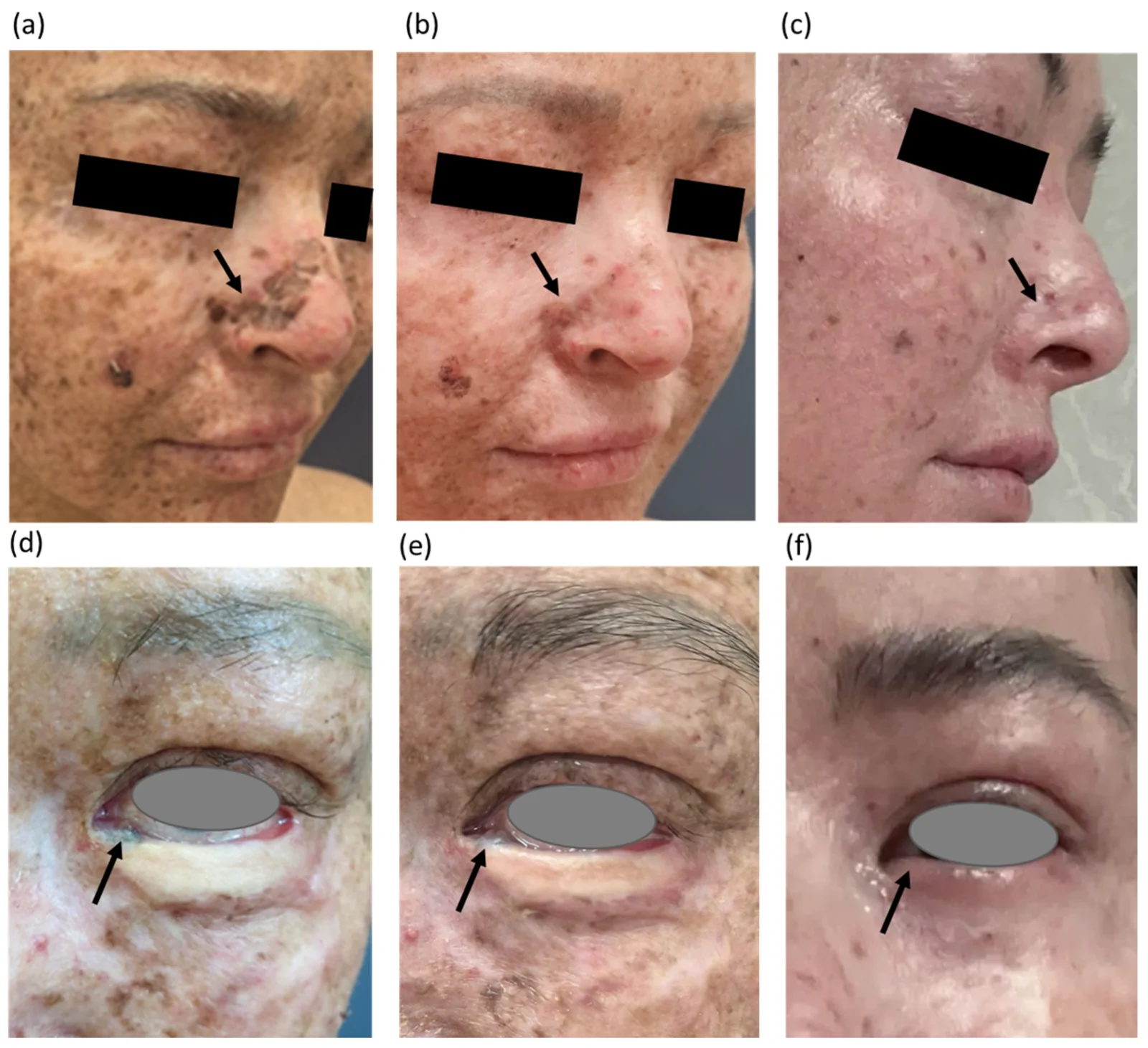
Patient ID7 before (**a**,**d**), after 2 months (**b**,**e**) and 7 months (**c**,**f**) of ICI therapy (pembrolizumab). Visible regression of nasal lentigo maligna melanoma; (**a**) compared to (**b**) and (**c**) (arrows). Regression of BCC lesion on the left lower eyelid: (**d**) compared to (**e**), and (**f**) (arrows).

Melanoma developed in three patients with XP. At the age of 22, patient ID7 was diagnosed with lentigo maligna melanoma (TisN0M0, stage 0) on the skin of the nasal dorsum, extending to the right nasal slope and ala (Figure 10a). The diagnosis was confirmed by punch biopsy and histopathological examination. Given the multiplicity of cutaneous malignancies and BCC progression during vismodegib therapy, anti-PD-1 immunotherapy with pembrolizumab was initiated (Figure 10). The primary indication was recurrent BCC not amenable to curative surgery or radiotherapy following progression on a Hedgehog pathway inhibitor (Figure 10d). The patient received pembrolizumab for two months, with substantial regression of the lentigo maligna melanoma (partial response by clinical assessment; no repeat biopsy has yet been performed) (Figure 10b) and a marked clinical response of the BCC lesion (Figure 10e). After 7 months, further improvement and near-complete regression of both malignant lesions were observed (Figure 10c,f).

In patient ID8, the following cutaneous melanomas were surgically excised:

- Four lentigo maligna melanomas, stage TisN0M0 (stage 0), located on the dorsum of the left hand (*n* = 2), right forearm (*n* = 1), and right auricle (*n* = 1);
- One nodular melanoma of the right lower extremity, T1bNxM0, Clark level II, Breslow thickness 1.0 mm, non-ulcerated;
- One nodular melanoma of the left lower extremity, T3aNxM0, Clark level IV, Breslow thickness 2.1 mm, non-ulcerated.

Patient ID11 had an ulcerated spindle cell pigmented melanoma with mild lymphocytic infiltrate, Clark level III invasion, Breslow thickness of 5 mm, and involvement of two lymph nodes (T4bN2bM0, stage IIIC). Surgical treatment was performed, and no mutations in *BRAF*, *NRAS*, or *KIT* were detected in the examined material. Given the high risk of disease progression, adjuvant immunotherapy with nivolumab was initiated in April 2023 and was ongoing until July 2026. The patient initially experienced diarrhea, a weight loss of 3–4 kg, and alopecia, which has persisted. Treatment was discontinued due to worsening cachexia and also because of stabilization of the patient’s condition and no evidence of disease. At the time of this report, the patient ID11 remains in complete remission.

### 2.5. Other Clinical Manifestations in XP Patients

Ocular abnormalities included dry eyes (ID4, ID9), lacrimation (ID6, ID7), corneal and conjunctival xerosis (ID3), keratoconjunctivitis or keratopathy (ID7, ID8, ID9, ID11), blepharitis (ID11), corneal opacity (ID7), and eyelid ectropion (ID7, ID8). Patients ID3, ID4, and ID7 were diagnosed with multiple benign nodules in both lobes of the thyroid gland at the ages of 10, 17, and 22, respectively. According to the Thyroid Imaging Reporting and Data System (TIRADS) classification, the lesions were classified as TIRADS III. Thyroid function tests revealed no abnormalities. No biopsy was performed, and the patients continue to be followed by an endocrinologist. Patients ID2 and ID11 developed severe valgus foot deformities. Scoliosis was noted in two patients (ID6 and ID9).

### 2.6. Genotype–Phenotype Correlations Revealed in the Present Study 

Different genetic defects in the *XPA*, *XPC*, *DDB2*, and *ERCC6* genes of the NER pathway were found in our cohort of 14 patients. Associations between clinical manifestations and genetic variants are presented in Table 3.

The small number of patients did not allow us to identify specific genotype–phenotype patterns, and these findings should therefore be considered preliminary. However, patients with pathogenic variants in *XPA*, compared with those with *XPC* variants, had an earlier onset of UV-associated skin damage (7 vs. 18 months), indicating higher photosensitivity. They also had a lower median age at first cancer (9.5 vs. 13 years). At the same time, the number of malignant skin tumors per patient was approximately twofold higher in patients with *XPC* variants than in those with *XPA* defects (5.9 vs. 2.8). Notably, all patients with pathogenic *XPA* variants had mild-to-moderate intellectual disability, whereas cognitive function was mostly preserved in the *XPC* group, although other neurological conditions (an asymptomatic brain cyst or AHDH) were observed in 25% of cases.

## 3. Discussion

Despite the low incidence of NER deficiency disorders in the general population, consanguinity significantly increases the risk of homozygous pathogenic variants in offspring. In our cohort, the parents of 10 of 14 patients were consanguineous. Furthermore, we identified a unique family with several consanguineous marriages in which carriers of pathogenic variants associated with XP and Cockayne syndrome were present, leading to the birth of three children affected by these rare disorders. This highlights the need for genetic counseling and carrier testing of relatives in families with confirmed cases of such diseases. It is also important to implement measures aimed at raising public awareness of the genetic nature of these conditions and the importance of family planning, including genetic testing of partners, especially in geographically or religiously isolated communities.

### 3.1. Frequency of XP-A, XP-C, and XP-E Complementation Groups in Different Populations

It is worth noting that the distribution of affected genes among XP complementation groups varies by region. We compared the frequency of pathogenic variants in the *XPA* and *XPC* genes in our study with published data (Table 4).

Pathogenic variants in the *XPC* gene are prevalent and account for more than 50% in most populations [5,36,37,38,39,40], while variants in the *XPA* gene are also not uncommon in Japan [43,44], China [37,45,46], Morocco [10], and Egypt [41,47]. Other complementation groups (XP-B, XP-D, XP-E, XP-G, and XP-V) are not rare and may account for 6% to 62% of cases [10,19,37,38,40,42]. In our cohort, *XPC* was the predominant gene (61%), while the frequency of *XPA* pathogenic variants accounted for 31%; a variant in *DDB2/XPE* was found.

### 3.2. Pathogenic/Likely Pathogenic Variants in the XPA Gene

The *XPA* gene, located at 9q22.33, encodes an essential nucleotide excision repair protein that functions in GG-NER and TC-NER. It binds to damaged DNA and contributes to lesion verification, while also helping to organize and stabilize the NER pre-incision complex, including TFIIH, RPA, and ERCC1–XPF. In GG-NER, initial lesion recognition is mediated primarily by XPC, which acts upstream of XPA [5,15,22].

The patients with XP-A exhibit a wide range of clinical phenotypes depending on the mutation site and, consequently, the residual functional XPA protein. The typical XP-A phenotype is characterized by both severe neurological impairment and extreme photosensitivity and is associated either with a complete splicing defect or profound alterations in the XPA protein structure as a result of mutations in exons 3, 4, and 5 [16,47]. Several scoring scales were used to describe neurological impairment in patients with XP-A and to analyze genotype–phenotype correlations in this group of patients. According to the classification suggested by Sagun et al. [16], which is based on the extent of neurological abnormalities at age 10 years, all our patients may be assigned to the intermediate disease group.

In our study, five pathogenic/likely pathogenic *XPA* variants were identified, including three homozygous and one compound heterozygous genotypes.

The homozygous missense mutation p.Cys105Arg (patient ID1) is located at the very beginning of the zinc-binding subdomain (aa 105–129) and was not previously described in the literature. The patient had mild intellectual disability (IQ of 53) and mild changes on brain MRI. The neurological symptoms progressed further, and at the age of 17 the patient was characterized by ataxia, tremor, and impaired coordination. Missense mutations in the *XPA* gene were described mainly in the zinc-binding domain [48] and were associated with milder neurological features. For example, a patient with two missense variants (p.Cys126Trp and p.Gln185His) had developmental delay (IQ of 50–85) by age 10 years and the onset of gait disturbance between ages 24–33 [16].

Another homozygous variant, p.Arg211*, results in a premature termination codon, predicted to cause truncation of the encoded protein (loss of part of the DNA-binding domain and the C-terminal domain that interacts with TFIIH) or absence of the protein due to nonsense-mediated decay (NMD). This molecular defect in patient ID3 was associated with more pronounced neurological and cognitive impairment (IQ 51 and marked gait disturbance). The variant c.631C>T (p.Arg211*) has been reported in the literature in individuals affected with XP from Japan [43] and China [45]. As mentioned earlier, truncating variants were associated with more severe neurological manifestations [16].

Patient ID2 carried two heterozygous pathogenic/likely pathogenic variants; he had the most severe neurological phenotype (IQ of 35) and noticeable brain MRI changes at age 10 years. The frameshift p.Leu182Cysfs*11 also leads to C-terminal domain truncation and disruption of XPA repair function. The *XPA* variant c.673+1G>A (rs2131393093) alters a conserved nucleotide located in a canonical splice site and is predicted by computational tools to affect mRNA splicing. This variant has been previously reported, but this is its first description in a patient with XP. The variant p.Leu182Cysfs*11 was novel and presented previously undescribed genomic alteration. It can be assumed that, in this case, the combination of splicing variant and premature termination of the protein chain led to even more severe neurological symptoms.

Another homozygous variant, *XPA* c.172+1G>T (rs1554703119), was found in patient ID11. The variant affects a donor splice site at the 5′ end of intron 1 and is classified as likely pathogenic according to computational predictors and the ACMG/AMP criteria. The patient had obvious neurological and cognitive impairment but was not fully examined.

**Figure 11:**
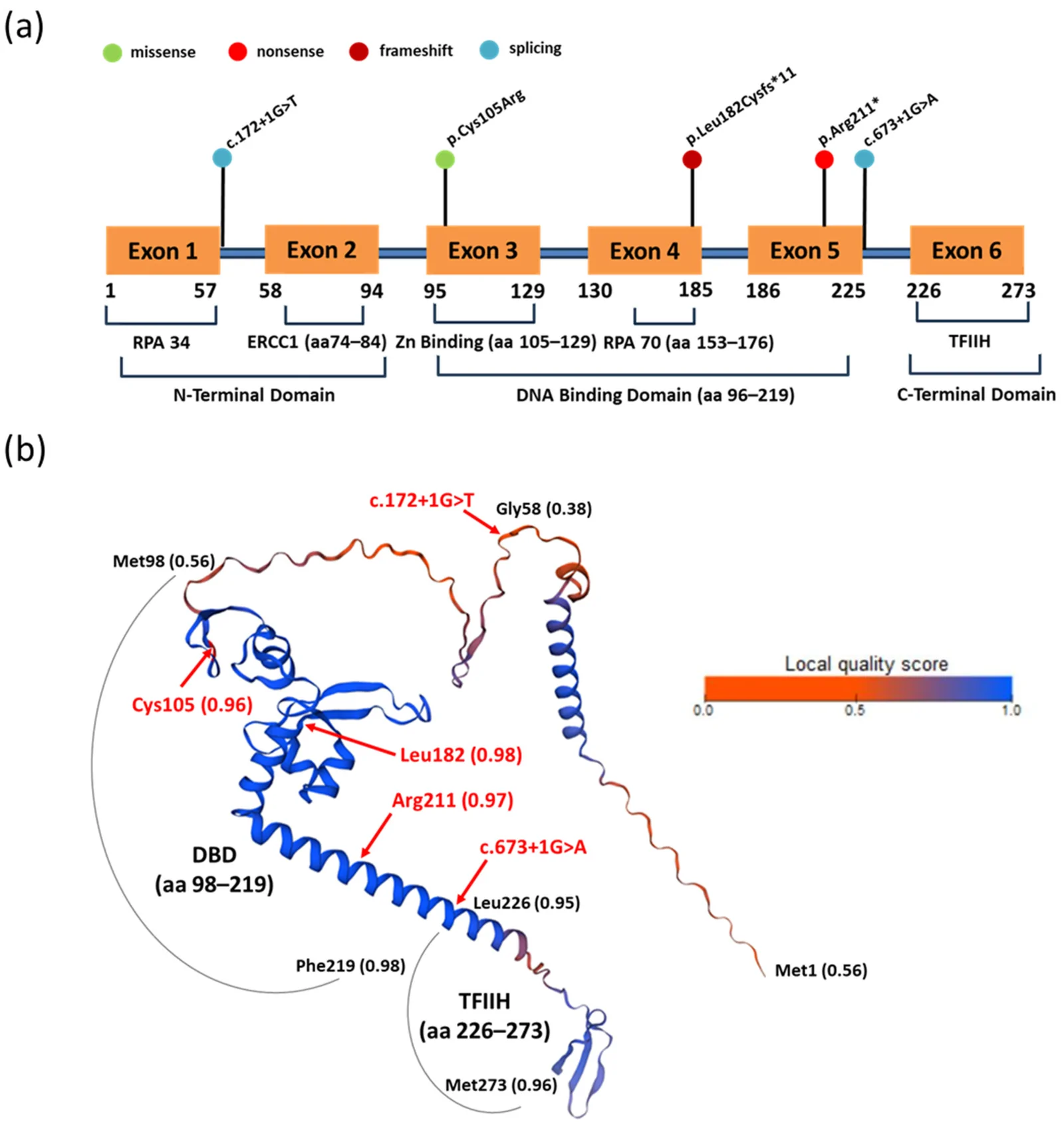
Distribution of pathogenic variants in the *XPA* gene (NM_000380.4). (**a**)—Exon structure of *XPA*, (**b**)—SWISS-MODEL structure of the human XPA protein (template P23025.1.A, Homo sapiens); GMQE = 0.81, QMEANDisCo = 0.62 ± 0.05. Local quality estimate (QMEAN) is shown on a color scale (0 to 1.0). Per-residue confidence scores (pLDDT) are given in parentheses (1 = high, 0 = low). Pathogenic variants are shown in red and indicated by arrows. RPA: replication protein A; TFIIH: transcription factor II H; DBD: DNA-binding domain; aa: amino acids.

The distribution of the variants identified along the *XPA* gene sequence is shown in Figure 11a. To visualize the localization of the identified variants in functional domains, a model of wild-type XPA protein was generated (Figure 11b and Figure S2) [49].

### 3.3. Pathogenic/Likely Pathogenic Variants in the XPC, DDB2 and ERCC6 Genes

**Figure 12:**
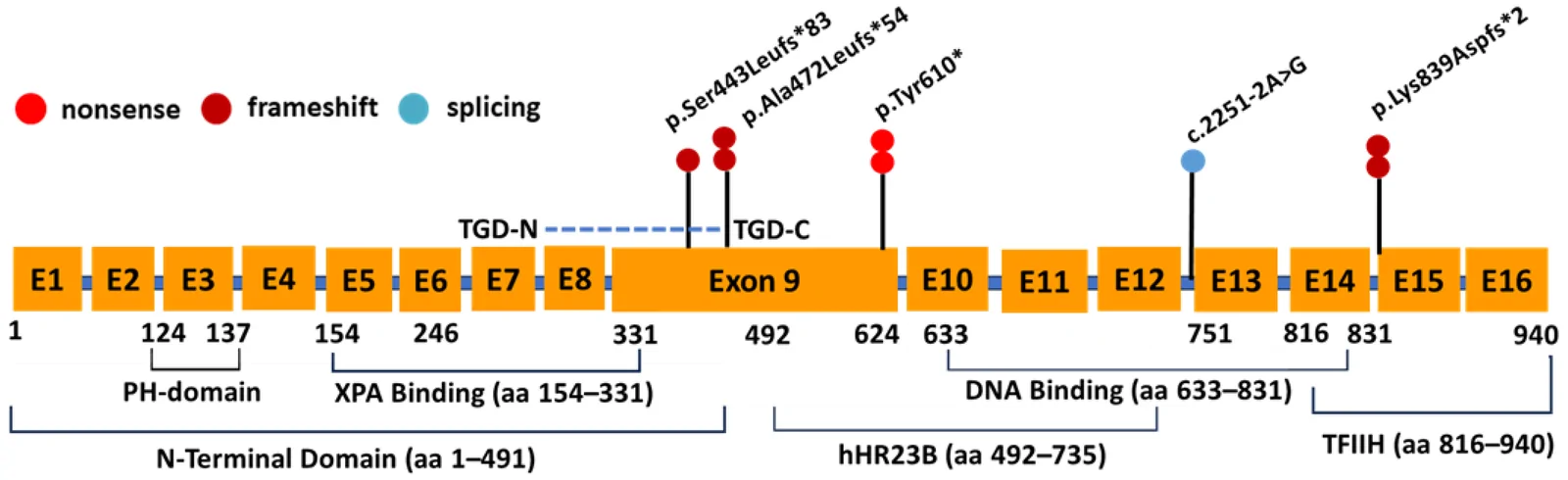
Exon structure of the *XPC* gene and distribution of pathogenic variants (NM_004628.5). E—exon, PH (Pleckstrin Homology)-domain binds with p62, hHR23B—human Homolog of Rad23B, TFIIH—transcription factor II H, aa—amino acids. The transglutaminase-like domain (TGD) includes N-terminal part (TGD-N) (aa 246–334) and C-terminal (TGD-C) (aa 426–495). The DNA-binding region composed of three β-hairpin domains (BHDs). BHD1 (aa 633–683), together with the TGD, anchors the protein and scans normal DNA duplexes. BHD2 (aa 684–741) assists in the initial non-specific DNA binding and searching process. BHD3 (aa 742–831) inserts a β-hairpin into the DNA duplex to flip out undamaged bases and form a stable recognition complex at lesion sites.

In total, we identified five pathogenic/likely pathogenic *XPC* variants in eight XP patients from six unrelated families. The *XPC* gene, mapped to chromosome 3p25.1, encodes a protein consisting of 940 amino acids. The distribution of *XPC* genetic variants within the gene is presented in Figure 12.

Patient ID7 carried a homozygous variant c.2251-2A>G, which affects an acceptor splice site in intron 12 of the *XPC* gene and is expected to disrupt RNA splicing. The neighboring nucleotide change c.2251-1G>C in the same splice site at the 3′ end of intron 12 has previously been observed in individuals with XP in multiple populations [39,40,50] and is considered a founder mutation.

The other *XPC* variants are novel and have not been previously described in XP patients; three of them are localized in exon 9 (Figure 12) and are predicted to affect the C-terminal part of XPC, which includes BHD1–3 and the TFIIH-binding site. Variants p.Ser443Leufs*83 (patient ID6) and p.Ala472Leufs*54 (patients ID8 and ID9) are frameshift variants that result in protein truncation (or NMD) and are classified as likely pathogenic by the ACMG/AMP. In addition, *XPC* p.Ala472Leufs*54 was found in two affected individuals from the same family.

In our sample, the *XPC* p.Tyr610* variant was detected in a homozygous state in two unrelated XP patients from Chuvashia (the Middle Volga region of European Russia) and in a heterozygous state in the healthy parents of these patients. We hypothesized that this variant may represent a founder mutation in this geographic region. Because haplotype analysis and additional population studies were beyond the scope of the present study, our assumption remains hypothetical.

A duplication of exon 14, leading to a frameshift and a premature stop codon (p.Lys839Aspfs*2), was found in two XP patients (ID12 and ID13) from the same family. This variant is predicted to affect the C-terminal part of the TFIIH-binding region.

Patient ID10 was found to carry the homozygous variant c.995G>A (p.Arg332His, R332H) in the *DDB2/XPE* gene. The human *DDB2* gene (locus 11p11.2) consists of 10 exons that encode a protein with a molecular mass of 48 kDa [51]. A characteristic feature of the DDB2 protein is the presence of WD-40 repeats in its C-terminal region, which form a circular, seven-bladed β-propeller structure that interacts with damaged DNA and DDB1. Previously identified pathogenic missense variants (R273H, D307Y, and L350P) are located primarily in the C-terminal half of the protein, within the β-propeller domain; these variants severely reduce cellular DDB2 levels, suggesting destabilization of the protein [52,53]. To assess the clinical significance of the variant R332H, we applied the following ACMG/AMP criteria: (1) a variant with a very low frequency in population databases (PM2); (2) homozygous occurrence in a proband with a recessive disorder who was born to consanguineous parents (PM3); and (3) a phenotype highly specific for the disease (PP4) (Table S3). The patient had an early manifestation of photosensitivity (at 1 month of age), but the first BCC was detected only at age 23. By age 30, the patient had developed 10 BCCs, which were successfully excised. Other phenotypic features are characteristic of the XP-E complementation group: mild-to-moderate skin pigmentation on the face and dyschromia of the upper and lower extremities. Despite a probable association of the variant R332H with the disease, the combined ACMG criteria support only a classification of uncertain significance (VUS). Further functional studies are required to clarify the variant pathogenicity.

Patient ID14 was found to be compound heterozygous for *ERCC6/CSB* pathogenic variants. The p.Gln102* variant results in a premature stop codon in the *ERCC6* gene and has been described in patients with Cockayne syndrome [54]. The other allelic variant p.Ser782Phefs*29 leads to a frameshift followed by protein truncation and is classified as likely pathogenic by ACMG. The patient had ophthalmological abnormalities that appeared during the first months of life, but severe neurological symptoms developed by the age of 3 years, likely indicating CS, Type II. No skin cancer was found by the age of 17 years.

### 3.4. Clinical Features of Patients with XP Compared with Global Data 

We compared selected clinical characteristics in our cohort and in other studies [40,42,55] (Table 5). Clinical characteristics depend significantly on the representation of different complementation groups and the age of the patients; therefore, we selected cohorts in which patients with *XPC* variants accounted for more than 50% of the total.

Overall, all the studies reviewed show a high rate of consanguinity (ranging from 63% to 94%) and a similar mean age at the onset of initial photosensitivity symptoms in the XP-C group (1.4–2.5 years). Most studies identify BCC as the most frequent type of skin cancer (72–86%), while melanoma is the least common (4–38%); however, its incidence can be notably higher (up to 36%) when older patients are included in the study cohort [40]. It should also be noted that some studies report an inability to perform histological examinations to confirm the tumor type, which introduces uncertainty into the clinical characterization of these patients.

The literature highlights patients with XP-A, XP-B, XP-D, XP-F, and XP-G as being the most prone to photosensitivity [42,56]. In our cohort, patients with XP-A exhibited earlier reactions to sun exposure and pigmentary changes, which may have prompted earlier implementation of sun-protection measures. Nevertheless, in our study, patients with XP-A developed their first skin cancer at an earlier age; however, patients with XP-C generally developed a higher number of tumors. The patients with XP-C experienced more severe disease, reflecting the critical role of the XPC protein in global genome repair; loss of this function leads to widespread mutagenesis and more aggressive cancer development. Meanwhile, patients with XP-A characteristically develop severe neurological manifestations, driven by the pivotal role of XPA in neuronal TC-NER [16,17]. In our study, all patients with pathogenic variants in the *XPA* gene exhibited varying degrees of intellectual disability. These neurological impairments are often associated with organic brain damage, which was observed on MRI scans in two of our patients.

### 3.5. Xeroderma Pigmentosum: Diagnosis and Management

Genetic testing should be performed as soon as XP is suspected, as it enables assessment of disease severity and possible extracutaneous abnormalities, given the known genotype–phenotype correlations [42,56]. Once the genotype is established, the patient can be referred for neurological and/or psychiatric evaluation, as well as brain MRI, to promptly address any potential abnormalities.

For patients with XP, the cornerstone of management is strict lifelong photoprotection against UV radiation, which should begin as early as possible. A key piece of evidence supporting this is a study of two sisters with an identical mutation in the *XPA* gene, where earlier rigorous sun protection allowed them to substantially increase their cancer-free period of life and to reduce the severity of neurological complications [57].

Complete avoidance of direct sunlight during daytime hours is essential, and when outdoor exposure is unavoidable, comprehensive protection should be employed, including sunscreen with SPF 50+ and high UVA protection, tightly woven protective clothing with long sleeves, gloves, wide-brimmed hats, and UV-blocking glasses or visors. It is important to note that UV radiation penetrates window glass and is emitted by certain types of lamps; therefore, installation of UV filters on windows and the use of safe light sources are also recommended indoors [58]. Furthermore, the use of gel nail polish is strongly discouraged for patients with XP, as its polymerization requires exposure to UV lamps, which constitute a direct source of damaging radiation [59]. Such stringent restrictions impose a considerable psychosocial burden on patients and their families, necessitating not only medical care but also psychological support [60].

According to the literature, thyroid nodules occur in more than 50% of patients with XP, with a mean age at onset greater than 20 years [61], and isolated cases of thyroid cancer have also been reported [62], predominantly in patients with pathogenic variants in the *XPC* gene. In our cohort, three patients with XP (23%) had nodules in both lobes of the thyroid gland at 10, 17, and 22 years of age, respectively; one was XP-A and two were XP-C. Given the wide range of extracutaneous manifestations, the management of patients with XP requires a multidisciplinary approach involving specialists from various fields.

Malignant neoplasms are a major factor reducing the quality and life expectancy of patients with XP; skin cancer is the most common of these, but there is also a wide range of internal malignancies [20,56,62]. Our data demonstrate a high risk of developing all types of malignant skin tumors (BCC, SCC, BSC, melanoma) in patients with XP as early as childhood and are consistent with global statistics. Furthermore, we present a unique case of multiple melanomas in an adolescent with the development of four primary melanomas in situ and two nodular melanomas over a 6-year follow-up period. Similar reports are rare and typically involve a later onset of the first melanoma [63].

For the treatment of patients in our cohort, surgical removal of lesions suspected of being malignant skin tumors, followed by histological confirmation, was the primary treatment approach. Targeted therapy and immunotherapy were required only in cases of locally advanced BCC or SCC, with significant clinical benefit. Notably, patients who received immunotherapy also showed an unexpected improvement in skin condition, manifested by a reduction in lentigines.

The literature describes cases of using targeted therapy and ICI therapy in patients with XP (Table S4). Regarding targeted therapy with Hedgehog signaling pathway inhibitors, six cases were found, including two children with palliative status who were only 8 years old [30,64,65,66,67,68]. In five cases, patients took vismodegib, and one patient took sonidegib. In almost all cases, pronounced side effects were noted: alopecia, myalgia, dyspeptic disorders, headache and amenorrhea [30,64,66]. However, in an 8-year-old patient who received vismodegib for 5 months, no impairment of bone development was observed [68]. In two patients, a complete clinical response was observed after 25 and 24 months of targeted therapy, respectively, and this response was maintained after treatment completion [65,68]. In addition, Jiménez-Sánchez et al. [65] noted a decrease in the severity of lentigines during the course of treatment. However, the effectiveness of targeted therapy in patients with XP remains controversial. In one case, despite significant tumor shrinkage, disease progression was observed after 18.5 months [30], while another patient was diagnosed with locally advanced SCC after a year of treatment with vismodegib [64].

This is consistent with our results: two patients (ID7 and ID8) who received Hedgehog signaling pathway inhibitors initially showed a complete response to treatment, but the therapy was discontinued due to significant side effects (alopecia, amenorrhea, and vomiting). During this treatment, new foci of BCC and melanoma also appeared in patient ID7, and multiple SCC lesions in patient ID8.

In worldwide data, ICI therapy was prescribed in 30 patients with XP due to melanoma (*n* = 11), SCC (*n* = 12), angiosarcoma (*n* = 3), basosquamous carcinoma (*n* = 1), non-small cell lung cancer (*n* = 1) with co-existing melanoma and SCC (*n* = 1), and sarcomatoid carcinoma with SCC and melanoma (*n* = 1) (Table S4). [29,64,66,69,70,71,72,73,74,75,76,77,78,79,80,81,82,83,84]. The median age of treatment initiation was 50,2 years (6–63), and there were seven pediatric patients (<18 years old). A total of 27 patients received pembrolizumab (*n* = 16), nivolumab (*n* = 8), or cemiplimab (*n* = 3) in monotherapy. One patient was first treated with pembrolizumab and then with nivolumab in monotherapy [79]. Combination (pembrolizumab and ipilimumab) was prescribed in one case [73]. Also, one patient consequently received nivolumab in monotherapy, nivolumab in combination with cetuximab and nivolumab in combination with ipilimumab [29,72]. The side effects were presented as dermatitis, rash, edema, myalgia, arthralgia, back pain, diarrhea, colitis, constipation, vomiting, elevated liver enzymes, hypothyroidism, fatigue, encephalitis, cough, adrenal insufficiency, fever, neutropenia, anemia and acute kidney injury [29,64,69,72,78,79,82,84]. All patients were alive; new foci of cancer were identified in six cases during or shortly after immunotherapy. In one case, progression (brain metastasis of angiosarcoma) was detected during a 3-month break after immunotherapy [79]. Another case was reported where a patient with an *XPC* variant who developed unresectable cutaneous SCC on his face showed a remarkable response to ICI, accompanied by an unexpected resolution of the lentigines. This rapid and sustained fading of all the patient’s lentigines was documented through three-dimensional whole-body imaging follow-up and persisted throughout therapy [84].

In our data, three patients received ICI for management of melanoma (*n* = 2), BCC (*n* = 1) and SCC (*n* = 1). Two patients pursued treatment without a new malignancy or side effects. One patient (ID11) stopped it due to cachexia and stabilization of the disease. In two patients (ID7 and ID8), a substantial fading of lentiginous pigmentation was also noticed during treatment.

However, the treatment of patients with XP, especially children, remains challenging and requires a multidisciplinary approach and careful lifelong monitoring.

### 3.6. Limitations of the Study

This study has several limitations. First, the small sample of patients (*n* = 14) and the limited number of observations used to assess the efficacy of targeted therapy (*n* = 2) and immunotherapy (*n* = 3) reduce the statistical power of the study. Second, the retrospective, single-center design and the variable follow-up durations across patients may have introduced bias and limited the uniformity of clinical data collection. Third, the lack of standardized criteria for assessing responses to systemic therapy, including immunotherapy and targeted agents, complicates direct comparisons of treatment outcomes. Fourth, the hypothesis of a founder effect in unrelated patients carrying the *XPC* p.Tyr610* variant who live in the same geographic region (Chuvashia) requires additional studies in a larger cohort of variant carriers and healthy residents of this region; such studies could not be conducted because of the limited availability of the necessary biomaterial. Fifth, the pathogenicity of the *DDB2* variant identified in one XP patient in homozygous state has not yet been proved conclusively, and its role in the pathogenesis of the disease remains uncertain. In this study, we were also unable to assess the preventive measures taken by the patients’ relatives to protect a child with obvious photosensitivity from the sun. Finally, the small cohort size, genetic heterogeneity, and incomplete clinical information in several cases limit the possibility of reliable genotype–phenotype correlations. However, given the orphan status of NER-related disorders, particularly xeroderma pigmentosum, the data presented are of considerable clinical significance and contribute to the global understanding of these conditions.

## 4. Materials and Methods

### 4.1. Patients

The study enrolled 14 patients: 13 with xeroderma pigmentosum (XP) and one with Cockayne syndrome (CS), including seven males and seven females, with ages ranging from childhood to young adulthood and a median age of 16.5 years (range 6–30). All patients were followed at the N.N. Blokhin National Medical Research Center of Oncology, Ministry of Health of the Russian Federation, between 2019 and 2026, with a median follow-up of 3 years (range 1–6) (Table S1). Written informed consent for participation in this study and for the publication of any potentially identifiable images was obtained from all patients. For each patient, the following data were collected: ethnicity, pedigree, phenotype description, medical history, dermatological, ophthalmological, and neurological examination reports, histological findings (if applicable), and information on anticancer treatment received. Clinical photographs demonstrating the response to treatment were taken using automated exposure. The images have not been altered, and no selective correction has been applied to them.

All patients underwent targeted next-generation sequencing (NGS) with a cancer-associated gene panel; for family members of patient ID12, trio whole-genome sequencing was additionally performed. The identified duplication was also screened by agarose gel electrophoresis in relatives of the patients, including patient ID13. Genomic DNA was isolated from peripheral blood lymphocytes using the HiPure Blood DNA Mini Kit (Magen Biotechnology Co., Guangzhou, China) according to the manufacturer’s protocol.

### 4.2. Next-Generation Sequencing

#### 4.2.1. Panel Sequencing

For library preparation, a hybridization-based selective enrichment method was employed using biotinylated probes targeting the coding regions of 415 genes associated with cancer development, including the following NER-related genes: *XPA*, *ERCC3*, *XPC*, *ERCC2*, *DDB2*, *ERCC4*, *ERCC5*, *POLH*, and the *ERCC6* and *ERCC8* genes associated with Cockayne syndrome (Table S2). The panel was designed using the HyperDesign online service (Roche, Basel, Switzerland) and based on a list of core cancer-associated genes derived from the commercial TruSight Hereditary Cancer Panel (Illumina, San Diego, CA, USA; https://www.illumina.com/products/by-type/clinical-research-products/trusight-cancer-hereditary.html#tabgroup-3-tab3, accessed on 31 January 2020), which was optimized for research purposes using data from the OMIM database (An Online Catalog of Human Genes and Genetic Disorders; https://omim.org/). The list of NER genes associated with the development of xeroderma pigmentosum, current as of August 2026, is available at https://omim.org/phenotypicSeries/PS278700, accessed on 20 August 2026. The panel included all of these genes except *GTF2H4*, whose association with xeroderma pigmentosum was first described in 2025 [3]. Libraries were prepared using the KAPA HyperPrep Kit (Roche, Basel, Switzerland), and hybridization was performed with the KAPA HyperCap Workflow v3.0 kit (Roche, Basel, Switzerland). Sequencing was carried out on a NextSeq2000 instrument (Illumina, San Diego, CA, USA) using the P1 300-cycle kit (Illumina, San Diego, CA, USA). The average coverage was 200–250×, with ≥90% of the target region covered at ≥50×, and a quality score of ≥99.9%.

Sequencing quality was assessed using FastQC. Read pairs were aligned to the reference genome (hg38) using the BWA-MEM2 algorithm. Duplicate reads were identified and removed using Picard MarkDuplicates. Base quality scores were recalibrated, and genetic variants were identified using Genome Analysis Toolkit (GATK) tools, specifically BQSR for score recalibration and HaplotypeCaller for variant calling. Filtering was based on total read depth (DP) ≥ 20×, quality score (QUAL) ≥ 30, and alternative allele depth (AD) ≥ 5. For heterozygous variants, a variant allele frequency (VAF) threshold of ≥30% was applied, whereas for homozygous variants, a VAF ≥ 90% was required. To minimize false-positive calls, standard GATK hard filters (QD < 2.0, FS > 60.0, MQ < 40.0) were applied, and variants located in low-complexity regions or homopolymer stretches were excluded.

Variant classification followed the ACMG/AMP 2015 guidelines for the interpretation of sequence variants [85] and ClinGen recommendations (https://www.clinicalgenome.org/tools/clingen-variant-classification-guidance/, accessed on 25 August 2026). To assess the pathogenicity of the identified variants, we used a comprehensive semi-automated workflow. Initial assessment and baseline evidence code generation were performed with the automated interpretation platform Varsome (https://varsome.com/, accessed on 20 September 2026), which provides a detailed breakdown of ACMG/AMP criteria and documents the underlying scoring algorithms. After the automated pipeline, all identified variants underwent manual curation and precise clinical re-evaluation to incorporate case-specific data. For variants detected in a homozygous or compound heterozygous state under an autosomal recessive inheritance model, the PM3 criterion was applied manually: PM3_Moderate for homozygotes from non-consanguineous marriages and for confirmed trans-heterozygotes (with the second allele classified as pathogenic or likely pathogenic), and PM3_Supporting for homozygotes from consanguineous marriages. The PP4_Supporting criterion was applied manually in all cases to account for the high clinical specificity of the patients’ phenotypes for the respective hereditary syndromes. This combined approach enabled precise, nuanced refinement of variant classifications (including upgrading initial VUS to Likely Pathogenic) in strict accordance with clinical data. The complete variant classification algorithm is described previously [86].

#### 4.2.2. Whole Genome Sequencing (WGS)

WGS was performed by the National Genetic Initiative “100,000 + Me” (NGI). Library construction was carried out using a PCR-free protocol on MGI sequencing platforms, following the standard guidelines. Paired-end sequencing (2 × 150 bp) was subsequently performed on a DNBSEQ-T7 genetic analyzer (MGI, Shenzhen, China), yielding an average genome coverage of 34x. The resulting reads were aligned to the human reference genome (hg38) using BWA version 0.7.17-r1188. Variant calling was conducted with DeepVariant. Copy number variations (CNVs) were assessed using Manta (v.1.6.0). Variant annotation was carried out using the certified software NGS-DATA-OMICS (registration number 2021662113) [87]. Read quality was evaluated with FastQC (v.0.11.9). Identified genetic variants were named according to HGVS nomenclature (v.21.1.2). Population allele frequencies were obtained from the 1000 Genomes Project and gnomAD (v.4.1.1). Clinical significance of the variants was assessed using ACMG guidelines [85].

### 4.3. Agarose Gel Electrophoresis

To detect the 245 bp duplication, we performed PCR using primers flanking the duplication boundaries (forward primer: AGTGATAGTCTGCGGGGTGG, reverse primer: TCGGCAGAATCTGGGGAGGA). The primers were designed using Primer-BLAST (https://www.ncbi.nlm.nih.gov/tools/primer-blast/, accessed on 25 May 2026) and amplified a sequence 447 bp in length in wild-type DNA. The PCR mixture (final volume 25 μL) consisted of 1× PCR-Buffer-B for Taq DNA polymerase; 2 mM MgCl2; 1 unit SynTaq DNA polymerase; 0.2 mM dNTPs; 0.04 μM of forward and reverse primers; and 20–30 ng DNA. All PCR reagents were produced by SYNTOL (Moscow, Russia), and primers were synthesized by Evrogen (Moscow, Russia). PCR was performed on a T100 Bio-Rad thermocycler (Hercules, CA, USA). The PCR cycling conditions were as follows: 3 min at 95 °C, followed by 35 cycles of 30 s at 95 °C, 30 s at 60 °C, 30 s at 72 °C, and a final extension at 72 °C for 2 min. Approximately 100–200 ng of the resulting PCR product was mixed with 4x Gel Loading Dye, Blue (Evrogen, Moscow, Russia) prior to loading onto a 2% agarose gel. Additionally, prior to gel casting, we added ethidium bromide to the agarose gel at a final concentration of 0.5 μg/mL for nucleic acid visualization. Electrophoresis was performed in 1× TAE buffer at 80 V for 120 min using a PowerPac Basic power supply (Bio-Rad, Hercules, CA, USA) and a horizontal SE-1 electrophoresis chamber (Helicon, Moscow, Russia). A 50+ bp DNA Ladder (Evrogen, Moscow, Russia, https://evrogen.ru/kit-user-manuals/DNA_Ladder_NL103.pdf, accessed on 28 July 2026), consisting of 11 fragments ranging from 50 to 700 bp, was used as a size marker. Following electrophoresis, the gel was directly visualized using a GE Typhoon FLA 9500 laser scanner (GE Healthcare, Danderyd, Sweden) with a 532 nm excitation laser and a 600 nm long-pass emission filter for ethidium bromide detection.

### 4.4. Homology Modeling

The three-dimensional structure of the XPA protein was generated by homology modeling using the SWISS-MODEL web server (Swiss Institute of Bioinformatics, Biozentrum, University of Basel, Switzerland) [49]. The target protein sequence (P23025.1.A, DNA repair protein complementing XP-A cells, Homo sapiens) was submitted to the server for automated template search against the SWISS-MODEL Template Library (SMTL) using BLAST [88] and HHblits [89]. The best-scoring template was selected based on the Global Model Quality Estimation (GMQE) score. Model building was performed using the ProMod3 comparative modeling engine integrated into the SWISS-MODEL pipeline. The quality of the resulting model was assessed using the QMEAN composite scoring function, which provides both global and per-residue quality estimates. Structural visualization and analysis were performed using the interactive tools available within the SWISS-MODEL web interface (https://swissmodel.expasy.org, accessed on 28 September 2026).

## 5. Conclusions

The study provides a comprehensive clinical and genetic characterization of a Russian cohort of patients with xeroderma pigmentosum (XP) and Cockayne syndrome, representing the first detailed report from this population. Our findings demonstrate that XP-C is the predominant complementation group among Russian patients with XP (61%), with a spectrum of pathogenic variants including both previously described and novel mutations. The identification of a recurrent *XPC* p.Tyr610* variant in two unrelated patients from the Chuvash Republic suggests a possible founder effect, warranting further population-based studies.

Consistent with the global literature, patients with *XPA* pathogenic variants exhibited earlier disease onset, more severe neurological impairment, and greater intellectual disability compared to those with *XPC* variants. In contrast, patients with affected *XPC* presented with a higher tumor burden and later age at symptom onset, reflecting the distinct roles of these proteins in nucleotide excision repair pathways.

We also present valuable real-world experience with systemic therapies in patients with XP. Both Hedgehog pathway inhibitors (vismodegib, sonidegib) and immune checkpoint inhibitors (pembrolizumab, nivolumab) demonstrated clinical efficacy in patients with advanced or multiple skin cancers, with some patients achieving complete responses. Importantly, immunotherapy was associated with an unexpected but clinically meaningful reduction in lentigines and photodamage, an observation that warrants further investigation.

The management of XP requires a multidisciplinary approach involving dermatologists, oncologists, neurologists, ophthalmologists, and psychologists. Strict photoprotection remains the cornerstone of care, and patients must be counseled to avoid not only sunlight but also artificial UV sources, including UV nail polish dryers. The psychosocial burden of these lifelong restrictions necessitates ongoing psychological support for patients and their families.

Despite the inherent limitations of a small, single-center cohort, our data contribute to the global understanding of XP by expanding the mutational spectrum, delineating genotype–phenotype correlations, and providing practical insights into the use of modern systemic therapies in this orphan disease. Larger, collaborative studies are needed to establish standardized treatment protocols and further elucidate the mechanisms underlying variable clinical expressivity in XP.

An earlier version of this work has been previously deposited as a preprint [90].

## Figures and Tables

**Table 1:** Clinical characteristics of patients. (BCC—basal cell carcinoma, SCC—squamous cell carcinoma, BSC—basosquamous carcinoma, ADHD—Attention Deficit Hyperactivity Disorder), DD—developmental delay, Pembro—pembrolizumab, CM—consanguineous marriage. (*)—age at the onset of follow-up, age at the present date. (**)—region of current inhabitance.

ID	Sex, Age * (Years)	Region ** (Ethnicity)	Gene	CM	First Symptom (Age, Month)	Cancer (Age, Years)	Neurologic	Ocular	Other	Treatment (Months)
ID1	F, 11, 16	North Caucasus (Avar)	*XPA*	Yes	Photodermatitis (6)	SCC (10), BCC (15), BCC (16)	Intellectual disability (mild), ataxia	No	No	Surgery
ID2	M, 10, 11	Central Russia (Slavic)	*XPA*	No	Photodermatitis (8)	BCC (9)	Intellectual disability (moderate)	No	Gothic palate, valgus foot deformity	Surgery
ID3	F, 9, 14	North Caucasus (Lak people)	*XPA*	No	Photodermatitis, lacrimation (2)	SCC (8)	Intellectual disability (mild-to-moderate), ataxia	Corneal- conjunctival xerosis	Coarse facial features, short neck, micrognathia, thyroid nodules	Surgery
ID4	F, 14, 17	Chuvashia (Slavic)	*XPC*	No	Lentigo (18)	SCC, BCC (11), BCC+ SCC (12), BSC (15), SCC (17)	No	Dry eyes	Thyroid nodules	Surgery
ID5	M, 5, 6	Chuvashia (Slavic)	*XPC*	No	Lentigo, xerosis cutis (24)	SCC (5)	ADHD	No	No	Surgery
ID6	F, 13, 14	North Caucasus (Avar)	*XPC*	Yes	Lentigo, photophobia (18)	BCC (13)	No	Lacrimation, photophobia	Scoliosis	Surgery
ID7	F, 18, 23	South Siberia (Kazakh)	*XPC*	Yes	Lentigo (24)	BCC (18), SCC (20) melanoma (22)	Cyst of the right hemisphere	Lacrimation, conjunctivitis, corneal opacity, ectropion	Thyroid nodules	Surgery, vismodegib (40), Pembro (2)
ID8	F, 14, 20	South Russia (Crimean Tatar)	*XPC*	Yes	Lentigo (18)	SCC (13), melanoma (13), BCC (17), SCC (19)	No	Ectropion, conjunctivitis, keratopathy, photophobia	No	Surgery, vismodegib (7), sonidegib (2) Pembro (11)
ID9	M, 12, 14				Photodermatitis (1)	SCC (14)	No	Dry eyes, keratopathy	Scoliosis	Surgery
ID10	F, 29, 30	North Caucasus (Avar)	*DDB2*	Yes	Photodermatitis (1)	BCC (23)	No	No	No	Surgery, radiation
ID11	M, 24, 27	North Caucasus (Dargin)	*XPA*	Yes	Photodermatitis (12)	BCC (22), melanoma (25)	Intellectual disability (moderate)	Kerato conjunctivitis, blepharitis	Foot deformity, underweight	Cryo- destruction, surgery, nivolumab (39)
ID12	M, 17, 21	Central Russia (Azerbaijani)	*XPC*	Yes	Photodermatitis (6)	BCC (13)	No	No	No	Surgery
ID13	M, 14, 15				Lentigo (24)	BCC (12)	No	No	No	Surgery
ID14	M, 13, 17		*ERCC6*	Yes	Nystagmus (1), cataract (6), Photodermatitis (12), DD (36)	No	Intellectual disability (severe), microcephaly	Nystagmus, cataract	Shortening of metacarpal bones, testicular hypoplasia, hearing loss, facial dysmorphism	No

**Table 2:** Variants in the *XPA*, *XPC*, *DDB2/XPE* and *ERCC6/CSB* genes identified in the study. F—family, ACMG—American College of Medical Genetics and Genomics, LP—likely pathogenic variant, P—pathogenic variant, hom—homozygote, het—heterozygote.

F	Patient	Gene	Nucleotide Change	Protein Change	Zygosity	ClinVar/ ACMG	dbSNP (rs)
1	ID1	*XPA*	c.313T>C	p.Cys105Arg	hom	–/LP	–
2	ID2	*XPA*	c.544_545insG	p.Leu182Cysfs*11	het	–/LP	–
			c.673+1G>A	–	het	LP/P	rs2131393093
3	ID3	*XPA*	c.631C>T	p.Arg211*	hom	P/P	rs149226993
4	ID4	*XPC*	c.1830G>T	p.Tyr610*	hom	–/LP	–
5	ID5						
6	ID6	*XPC*	c.1327del	p.Ser443Leufs*83	hom	–/LP	–
7	ID7	*XPC*	c.2251-2A>G	–	hom	P/P	rs1553604570
8	ID8	*XPC*	c.1413del	p.Ala472Leufs*54	hom	–/LP	–
	ID9						
9	ID10	*DDB2/XPE*	c.995G>A	p.Arg332His	hom	VUS/VUS	–
10	ID11	*XPA*	c.172+1G>T	–	hom	LP/LP	rs1554703119
11	ID12	*XPC*	c.2421_2514dup	p.Lys839Aspfs*2	hom	–/LP	–
	ID13						
11	ID14	*ERCC6/CSB*	c.304C>T	p.Gln102*	het	P/P	rs1339834464
			c.2344dup	p.Ser782Phefs*29	het	–/LP	-

**Table 3:** Summary of clinical characteristics in patients with variants in different genes (*n*—number of patients).

Characteristics	*XPA* (*n* = 4)	*XPC* (*n* = 8)	*DDB2* (*n* = 1)	*ERCC6* (*n* = 1)
Age at present date (years), median (range)	15 (11–27)	16 (6–23)	30	17
Age at first symptoms (months), median (range)	7 (2–12)	18 (1–24)	1	1
Age at first skin cancer (years), median (range)	9.5 (8–22)	13 (5–18)	23	N/A
BCC (or BSC), patients, *n* (%)	3 (75)	6 (75)	1(100)	0 (0)
SCC, patients, *n* (%)	2 (50)	5 (63)	0 (0)	0 (0)
Melanoma, patients, *n* (%)	1 (25)	2 (25)	0 (0)	0 (0)
Average number of tumors per one patient, *n*	2.8	5.9	10	0 (0)
Photosensitivity, *n* (%)	4 (100)	8 (100)	1 (100)	1 (100)
Neurological conditions, *n* (%)	4 (100)	2 (25)	0 (0)	1 (100)
Intellectual disability, *n* (%)	4 (100)	0 (0)	0 (0)	1 (100)
Impaired gait, *n* (%)	2 (50)	0 (0)	0 (0)	1 (100)
Structural brain anomalies, *n* (%)	2 (50)	1 (13)	0 (0)	1 (100)
Mental problems, *n* (%)	4 (100)	1 (13)	0 (0)	1 (100)
Epilepsy, *n* (%)	1 (25)	0 (0)	0 (0)	0 (0)
Ophthalmopathy, *n* (%)	2 (50)	5 (63)	0 (0)	1 (100)
Thyroid neoplasms, *n* (%)	1 (25)	2 (25)	0 (0)	0 (0)
Skeletal anomalies, *n* (%)	3 (75)	2 (25)	0 (0)	1 (100)

**Table 4:** Frequency of XP-A and XP-C complementation groups in different populations. Others include complementation groups XP-B, XP-D, XP-E, XP-G, and XP-V.

Study	Country, Origin (Number of Patients)	Age (Years) Median (Range)	Complementation Groups		
			XP-A, *n* (%)	XP-C, *n* (%)	Other, *n* (%)
Schäfer et al., 2013 [36]	Germany (16)	9.4 (2–19)	0 (0)	16 (100)	0 (0)
Sun et al., 2015 [37]	China (12)	7 (0–26)	3 (25)	4 (33)	5 (42)
Espi et al., 2018 [38]	Nepal (17)	15 (1–32)	1 (6)	15 (88)	1 (6)
Ijaz et al., 2019 [39]	Pakistan (9)	6 (3–12)	0 (0)	9 (100)	0 (0)
Santiago et al., 2020 [40]	Brazil (32)	30 (2–82)	0 (0)	22 (69)	10 (31)
Rabie et al., 2021 [41]	Egypt (36)	8 (0–32)	7 (19)	29 (81)	0 (0)
Abbassi et al., 2022 [10]	Morocco (40)	9.7 (1–33)	9 (23)	21 (52)	10 (25)
Sarasin, 2023 [19]	France (181)	26 (8–88)	13 (7)	118 (65)	50 (28)
	French (37)	–	1 (3)	13 (35)	23 (62)
	North Africa (48)	–	3 (6)	35 (73)	10 (21)
	Algeria (29)	–	2 (7)	22 (76)	5 (17)
	Tunisia (21)	–	0 (0)	17 (81)	4 (19)
	Morocco (22)	–	6 (27)	16 (59)	0 (0)
Baskurt et al., 2026 [42]	Turkey (69)	23 (2–54)	3 (4)	38 (55)	28 (41)
Present study, 2026	Russia (13)	16.5 (6–30)	4 (31)	8 (61)	1 (8)

**Table 5:** Clinical characteristics of XP patients from different populations.

Characteristic	Present Study (*n* = 13)	Santiago et al., 2020 (*n* = 32) [40]	Khalat et al., 2023 (*n* = 23) [55]	Baskurt et al., 2026 (*n* = 69) [42]
Median age, years	16.5 (6–30)	30.5 (2–82)	8 (2–35)	23 (2–54)
*XPC* variants	61%	69%	83%	55%
*XPA* variants	31%	0%	8%	4%
Consanguinity	69%	63%	94%	74%
Mean age at first symptoms in all cohorts (years)	1.0	2.5	1.7	5.2
Mean age at first symptoms in the *XPC* group (years)	1.4	1.6	1.7	2.5
Mean age of first skin tumor (biopsy) (years)	13	8.8	ND	16.4
Mean age of first skin tumor (biopsy) in the *XPC* group (years)	12.4	5.5	ND	12.2
BCC (patients, *%*)	77%	86%	9%	72%
SCC (patients, *%*)	54%	64%	30%	48%
Melanoma (patients, *%*)	23%	36%	4%	38%
Neurologic impairment	46%	3%	ND	14%
Ophtalmopathy	54%	56% (severe)	ND	57%

## Data Availability

A preprint version of this manuscript is available on Preprints.org [90]. The clinical and genetic data presented in this study are available within the article and its Supplementary Materials.

## Supplementary Materials

The following supporting information can be downloaded at: https://www.mdpi.com/article/10.3390/ijms27219889/s1.
