# Supplementary material for: Clinical Features, Genetics and Treatment in a Russian Cohort of Young Patients with Xeroderma Pigmentosum: A Single Center Experience
Source: Int J Mol Sci. 2026 Nov 4;27(21):9889. doi: 10.3390/ijms27219889 (PMC13764289; doi:10.3390/ijms27219889)
Supplement: Supplementary file 1 [file ijms-27-09889-s001.zip › Figure S1_Agarose electrophoresis.pdf]

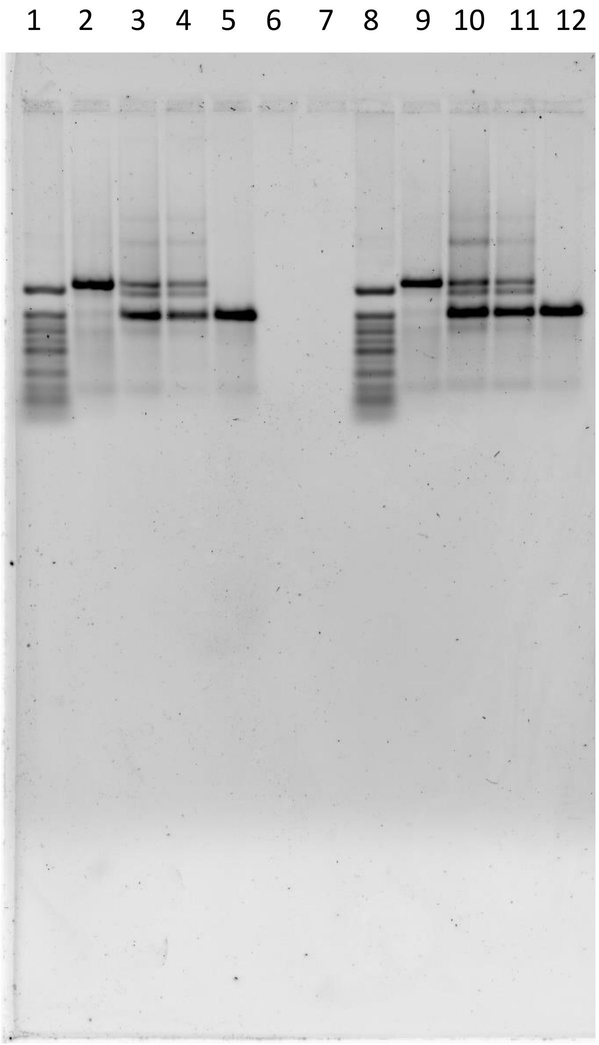

Figure S1. – Agarose gel electrophoresis of exon 14 PCR products (1 – DNA ladder; 2 – ID12, 3 – father of ID12; 4 – mother of ID12; 5 – wild-type control; 6, 7 – empty gel; 8 – DNA ladder; 9 – ID13, 10 – father of ID13; 11 – mother of ID13; 12 – wild-type control).
