# Supplementary material for: Clinical Features, Genetics and Treatment in a Russian Cohort of Young Patients with Xeroderma Pigmentosum: A Single Center Experience
Source: Int J Mol Sci. 2026 Nov 4;27(21):9889. doi: 10.3390/ijms27219889 (PMC13764289; doi:10.3390/ijms27219889)
Supplement: Supplementary file 1 [file ijms-27-09889-s001.zip › Figure S2. Homology modelling and quality assessment for wild-type XPA protein.pdf]

Figure S2. Homology modelling and quality assessment for wild-type XPA protein.

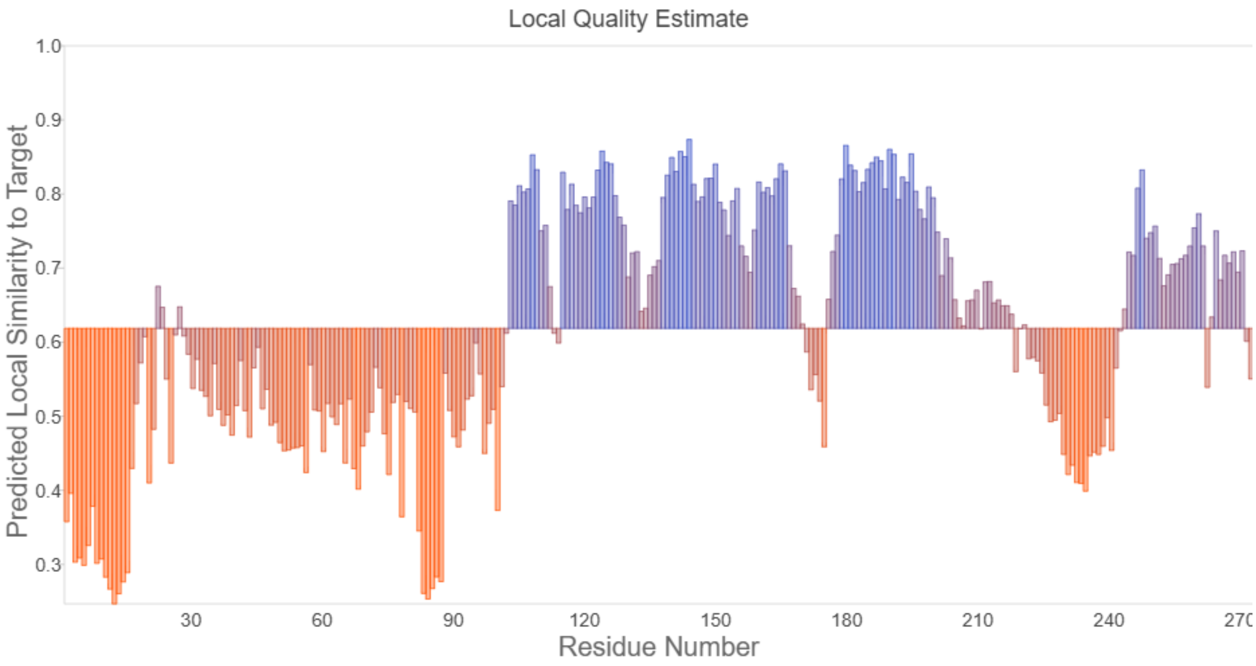

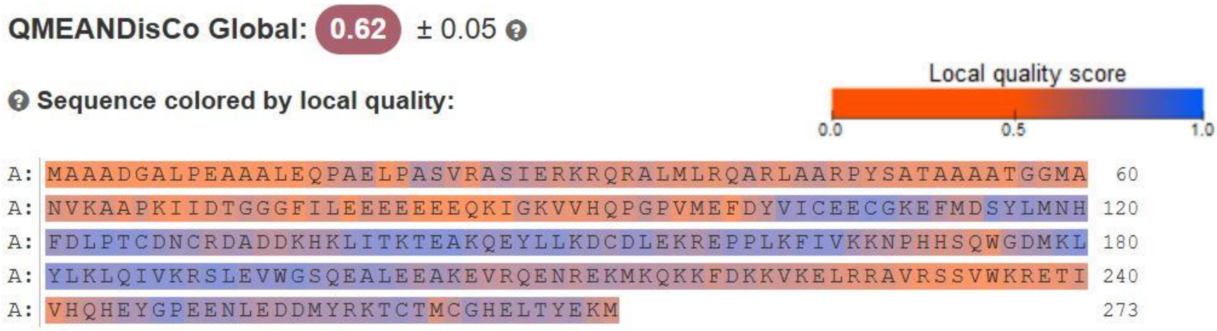

The model was generated via the SWISS-MODEL web interface [Waterhouse A, Bertoni M, Bienert S, Studer G, Tauriello G, Gumienny R, Heer FT, de Beer TAP, Rempfer C, Bordoli L, et al. SWISS-MODEL: homology modelling of protein structures and complexes. Nucleic Acids Res. 2018, 46(W1):W296-W303. doi: 10.1093/nar/gky427.] using template P23025.1.A (DNA repair protein complementing XP-A cells, Homo sapiens) with 100% sequence identity and coverage 1–273, indicating that template P23025.1.A matches the sequence of the modelled XPA protein across all 273 residues, without gaps or substitutions; the global model quality estimate GMQE was 0.81, indicating high expected accuracy of the build. In addition, QMEANDisCo (Qualitative Model Energy ANalysis with Distance Constraints is able to derive both global (i.e. for the entire structure) and local (i.e. per residue) absolute quality estimates on the basis of one single model) was calculated via the SWISS-MODEL service (https://swissmodel.expasy.org/qmean/):

the global score QMEANDisCo Global = 0.62 ± 0.05 corresponds to moderate model quality. Thus, the model is suitable for visualizing the overall protein fold and the approximate localization of variants, but it is not a high-quality model, and therefore fine conclusions about local conformation should not be drawn from it [Studer G, Rempfer C, Waterhouse AM, Gumienny R, Haas J, Schwede T QMEANDisCo - distance constraints applied on model quality estimation. Bioinformatics 36, 1765-1771. (2020) PIMD 31697312 doi: 10.1093/bioinformatics/btz828].

Quality was then assessed for individual amino acid residues. Per-residue quality was evaluated using the Local Quality Score — the per-residue QMEANDisCo estimate (from 0 to 1) reflecting how well the geometry and packing of a residue match those expected for real protein structures (the closer to 1, the higher the quality) — and Confidence (pLDDT) — a measure of the model's confidence in the localization of a residue (from 0 to 1), where high values correspond to structurally stable regions and low values to flexible or disordered regions. The assessment results are presented below.

**Mutation**	**Local Quality Score**	**Confidence (pLDDT)**
c.172+1G>T (Gly58)	0.57	0.38
p.Cys105Arg	0.87	0.96
p.Leu182CysfsTer11	0.83	0.98
p.Arg211Ter	0.62	0.97
c.673+1G>A (Leu226)	0.52	0.95
