# Supplementary material for: Clinical Features, Genetics and Treatment in a Russian Cohort of Young Patients with Xeroderma Pigmentosum: A Single Center Experience
Source: Int J Mol Sci. 2026 Nov 4;27(21):9889. doi: 10.3390/ijms27219889 (PMC13764289; doi:10.3390/ijms27219889)
Supplement: Supplementary file 1 [file ijms-27-09889-s001.zip › Table S4. Targeted and ICI treatment in XP patients.pdf]

Table S4. Targeted and ICI therapy in XP patients.

Article	Sex, age years (on the beginning therapy)	Gene	Malignant tumor	Type of targeted therapy (duration, months)	Complications	Outcomes
**Inhibitors of Hedgehog signaling pathway**						
Soura E et al, 2018 [30]	F, 21	ND	mBCC	Vismodegib (19 and 23 after interruption)	Alopecia, dysgeusia, muscle cramps, and amenorrhea.	Rapid progression after 18,5 months of target therapy
Rubatto M et al, 2022 [64]	F, 18*	ND	mBCC	Vismodegib (1)	Alopecia	Alive, locally advanced SCC after a year of treatment
Jiménez-Sánchez A et al, 2026 [65]	M, 68	ND	mBCC	Sonidegib (24)	ND	Alive
Boutros C et al, 2021 [66]	M, 33	*XPC*	Advanced BCC	Vismodegib (1 and 24 after interruption)	Diarrhea	Alive
Cordeiro MEC at al, 2023 [67]	M, 8	ND	mBCC	Vismodegib (1)	ND	Alive
Fife D et al, 2017 [68]	M, 8	ND	Advanced BCC of the nose skin	Vismodegib (25)	ND	Alive
Present, 2026	F, 18 (ID7)	*XPC*	mBCC	Vismodegib (40)	Alopecia, amenorrea	Alive
	F, 18 (ID8)	*XPC*	mBCC	Vismodegib (7) Sonidegib (2)	Alopecia, amenorrea, vomiting	Alive
**Immune checkpoint inhibitors**						
Dumas M et al, 2026 [29]	F, 24	*XPC*	Metastatic SCC	Pembrolizumab (12)	No	Alive

	M, 17	*XPC*	Basosquamous carcinoma, that evolved to metastatic SCC	Pembrolizumab (8)	No	Alive
	M, 15	*XPC*	Metastatic SCC	Pembrolizumab (6)	No	Alive
	F, 6**	*XPC*	SCC	Nivolumab in combination with cetuximab (3)	Neutropenia (grade 1), renal function alteration (grade 1)	Progression after 3 months and development of 2 melanomas after 9 months and 5 years of ICI initiation
			Metastatic melanoma	Nivolumab in combination with ipilimumab	No	Alive
Rubatto M et al, 2022 [64]	F, 19*	ND	Advanced SCC	Cemiplimab (5)	Diarrhea	Alive
Boutros C et al, 2021 [66]	M, 33	*XPC*	Cutaneous angiosarcoma of the left inner canthus	Nivolumab (18) after incomplete resection and chemotherapy	ND	Alive, remission persisting 41 months after last ICI cycle
Hauschild A et al, 2017 [69]	M, 51	*XPE*	Melanoma pT1bN2aM1c	Pembrolizumab (3)	Dermatitis	Alive
Deinlein T et al, 2017 [70]	F, 48	ND	Metastatic SCC	Pembrolizumab (2)	ND	Alive
Chambon F et al, 2018 [71]	F, 6**	*XPC*	Sarcomatoid carcinoma	Nivolumab (3)	ND	Alive
Salomon G et al, 2018 [72]	M, 17	*XPC*	Melanoma pT4bN0M1c	Pembrolizumab (3)	Vitiligo-like dermatitis	Aliva
Ameri AH et al, 2019 [73]	F, 18	*XPC*	Advanced SCC	Pembrolizumab (8)	ND	Alive

	M, 19	*XPE*	Advanced SCC	Pembrolizumab (3)	ND	Alive
	F, 20	*XPV*	Melanoma, advanced SCC	Ipilimumab Pembrolizumab (31)	ND	Alive
Steineck A et al, 2019 [74]	F, 7	*XPC*	Metastatic SCC	Pembrolizumab (4)	ND	Alive
Momen S et al, 2019 [75]	M, 32***	*XPC*	Cutaneous angiosarcoma	Pembrolizumab (3)	ND	Alive
Hennemann A et al, 2022 [76]	M, 58	*XPV*	Metastatic SCC	Pembrolizumab (15)	No	Alive
Şahin EA et al, 2022 [77]	M, 7	*XPC*	Reccuring SCC	Nivolumab (4)	No	Alive
Scheer V et al, 2022 [78]	F, 49	*XPC*	Superficial spreading melanoma	Nivolumab (12)	Rush	Alive
	M, 34	*XPD*	Metastatic melanoma	Nivolumab (10)	Hypothyroidism, elevated liver enzymes	Alive
	M, 63	*XPC*	Multiple primary melanomas	Nivolumab (4)	Encephalitis, depressed consciousness, colitis, fatigue, hypothyroidism, adrenal insufficiency, vomiting, abdominal pain, cough, fever, acute nontraumatic kidney injury, muscle atrophy, diarrhea	Alive, 3 BCC and 1 SCC occurred 3-50 months after last cycle
	M, 57	*XPC*	Metastatic melanoma	Pembrolizumab (24)	Fatigue, hypothyroidism	Alive, 1 SCC occurred during ICI treatment

Fernandez ER et al, 2023 [79]	F, 58	*XPC*	Metastatic amelanotic melanoma	Nivolumab (10)	Fatigue, hypothyroidism	Alive, 3 melanomas in situ occurred during ICI treatment
	F, 59	*XPC*	Metastatic NSCLC from left lower lobe mass	Pembrolizumab (12)	Rash, myalgia, arthralgia	Alive
	F, 35	*XPC*	Metastatic melanoma with unknown primary tumor	Pembrolizumab (1,5)<br>Nivolumab (1)	Rash, pruritus, elevated liver enzymes, fatigue, hot flashes, edema, myalgia, back pain, abdominal pain, constipation, cough	Alive, 1 BCC occurred 3 months after last ICI cycle
	M, 32***	*XPC*	Metastatic cutaneous angiosarcoma	Pembrolizumab (30)	No	Alive, during a 3-month break from ICI, the patient developed a brain metastasis. Ongoing treatment
	M, 62	*XPV*	Metastatic melanoma with unknown primary	Nivolumab (18)	Skin hypopigmentation	Alive, 1 invasive melanoma (pT2a) occurred 25 months after last ICI cycle
	M, 33	*XPC*	Metastatic melanoma with unknown primary tumor	Pembrolizumab (24)	No	Alive
Boziou M et al, 2023 [80]	F, 29	ND	Locally advanced SCC	Cemiplimab (18)	No	Alive
Zalaquett NG et al, 2024 [81]	F, 49	ND	SCC, T2N2cM0	Pembrolizumab (24)	No	Alive
Gambichler T et al, 2025 [82]	M, 7	*XPC*	Advanced SCC	Cemiplimab (8)	Intermittent fever, anemia	Alive
Proietti I et al, 2025 [83]	M, 26	ND	Metastatic melanoma	Nivolumab (6)	No	Alive

Brufau-Cochs M. et al., 2026 [84]	M, 54	*XPC*	SCC (face)	Pembrolizumab	Abrupt-onset type 1 diabetes mellitus (at month 18), a mild hepatitis (at month 24).	After 2 years of follow-up, the patient continues pembrolizumab with a sustained oncological response
Present, 2026	F, 18 (ID7)	*XPC*	Melanoma	Pembrolizumab (2)	No	Alive, treatment is going on
	F, 18 (ID8)	*XPC*	Advanced SCC	Pembrolizumab (11)	Dermatitis	Alive, treatment is going on
	M, 25 (ID10)	*XPA*	Melanoma	Nivolumab (39)	Cachexia	Alive

Abbreviations: BCC – basal cell carcinoma, ICI - immune checkpoint inhibitors, mBCC – multiple BCC, NSCLC – non-small cell lung cancer SCC – squamous cell carcinoma
** the treatment of this patient included Hedgehog inhibitors (mBCC) and then immune checkpoint inhibitors (locally advanced SCC)

** this patient was described in 2 publications (first cancer in one article [71] and further history of patient in the second one) [29]

*** this patient was also described in 2 publications (first cancer in one article and further history of patient in the second one) [75,79]

References

29. Dumas, M.; Réguerre, Y.; Robert, E.; Scalbert, C.; Bertolotti, A.; Miquel, A. Efficacy of immune checkpoint inhibitors in patients with xeroderma pigmentosum and advanced cutaneous squamous-cell carcinoma and literature review. Ann. Dermatol. Ve-nereol. 2026, 153, 103487. https://doi.org/10.1016/j.annder.2026.103487.

30. Soura, E.; Plaka, M.; Dessinioti, C.; Chasapi, V.; Stefanaki, C.; Antoniou, C.; Stratigos, A. Use of vismodegib for the treatment of multiple basal cell carcinomas in a patient with xeroderma pigmentosum. Pediatr. Dermatol. 2018, 35, e334–e336. https://doi.org/10.1111/pde.13610. PM ID: 30178564.

64. Rubatto, M.; Merli, M.; Avallone, G.; Agostini, A.; Mastorino, L.; Caliendo, V.; Barcellini, A.; Vitolo, V.; Valvo, F.; Fierro, M.T.; et al. Immunotherapy in Xeroderma Pigmentosum: A case of advanced cutaneous squamous cell carcinoma treated with cemi-plimab and a literature review. Oncotarget 2021, 12, 1116–1121. https://doi.org/10.18632/oncotarget.27966. Erratum in Onco-target 2022, 13, 985. https://doi.org/10.18632/oncotarget.28265..

65. Jiménez-Sánchez, A.; Delgado-Jiménez, Y.; Hernández-Marín, B.; Rodríguez-Jiménez, P. Treatment with sonidegib for multiple basal cell carcinomas in a patient with xeroderma pigmentosum. Actas Dermo-Sifiliográficas 2026, 117, 104628. https://doi.org/10.1016/j.ad.2026.104628.

66. Boutros, C.; Rouleau, E.; Majer, M.; Nikolaev, S.; Robert, C. Combination of targeted therapy and immune checkpoint blocker in a patient with xeroderma pigmentosum presenting an aggressive angiosarcoma and a recurrent non-resectable basal cell carcinoma. Eur. J. Cancer 2021, 150, 130–132. https://doi.org/10.1016/j.ejca.2021.03.022 .

67. Cordeiro, M.E.C.; Real, L.C.; Simoni, A.G.P. Xeroderma pigmentosum: Case report. Rev. Paul. Pediatr. 2023, 41, e2021390. https://doi.org/10.1590/1984-0462/2023/41/2021390.

68. Fife, D.; Laitinen, M.A.; Myers, D.J.; Landsteiner, P.B. Vismodegib Therapy for Basal Cell Carcinoma in an 8-Year-Old Chinese Boy with Xeroderma Pigmentosum. Pediatr. Dermatol. 2017, 34, 163–165. https://doi.org/10.1111/pde.13080.

69. Hauschild, A.; Eichstaedt, J.; Möbus, L.; Kähler, K.; Weichenthal, M.; Schwarz, T.; Weidinger, S. Regression of melanoma metastases and multiple non-melanoma skin cancers in xeroderma pigmentosum by the PD1-antibody pem-brolizumab. Eur. J. Cancer 2017, 77, 84–87. https://doi.org/10.1016/j.ejca.2017.02.026.

70. Deinlein, T.; Lax, S.F.; Schwarz, T.; Giuffrida, R.; Schmid-Zalaudek, K.; Zalaudek, I. Rapid response of metastatic cutaneous squamous cell carcinoma to pembrolizumab in a patient with xeroderma pigmentosum: Case report and review of the literature. Eur. J. Cancer 2017, 83, 99–102. https://doi.org/10.1016/j.ejca.2017.06.022.

71. Chambon, F.; Osdoit, S.; Bagny, K.; Moro, A.; Nguyen, J.; Réguerre, Y. Dramatic response to nivolumab in xeroderma pig-mentosum skin tumor. Pediatr. Blood Cancer 2018, 65, e26837. https://doi.org/10.1002/pbc.26837.

72. Salomon, G.; Maza, A.; Boulinguez, S.; Paul, C.; Lamant, L.; Tournier, E.; Mazereeuw-Hautier, J.; Meyer, N. Efficacy of an-ti-programmed cell death-1 immunotherapy for skin carcinomas and melanoma metastases in a patient with xero-derma pigmentosum. Br. J. Dermatol. 2018, 178, 1199–1203.

73. Ameri, A.H.; Mooradian, M.J.; Emerick, K.S.; Park, J.C.; Wirth, L.J.; Asgari, M.M.; Tsao, H.; Lawrence, D.; Sullivan, R.J.; Demehri, S. Immunotherapeutic strategies for cutaneous squamous cell carcinoma prevention in xeroderma pigmentosum. Br. J. Dermatol. 2019, 181, 1095–1097. https://doi.org/10.1111/bjd.18144.

74. Steineck, A.; Krumm, N.; Sarthy, J.F.; Pritchard, C.C.; Chapman, T.; Stacey, A.W.; Vitanza, N.A.; Cole, B. Response to Pem-brolizumab in a Patient With Xeroderma Pigmentosum and Advanced Squamous Cell Carcinoma. JCO Precis. Oncol. 2019, 3, 1–6. https://doi.org/10.1200/PO.19.00028.

75. Momen, S.; Fassihi, H.; Davies, H.R.; Nikolaou, C.; Degasperi, A.; Stefanato, C.M.; Dias, J.M.L.; Dasgupta, D.; Craythorne, E.; Sarkany, R.; et al. Dramatic response of metastatic cutaneous angiosarcoma to an immune checkpoint inhibitor in a patient with xeroderma pigmentosum: Whole-genome sequencing aids treatment decision in end-stage disease. Cold Spring Harb. Mol. Case Stud. 2019, 5, a004408. https://doi.org/10.1101/mcs.a004408.

76. Hennemann, A.; Collonge Rame, M.A.; Puzenat, E.; Ged, C.; Harbon, S.; Aubin, F.; Nardin, C. Efficacy of pembrolizumab in a patient with xeroderma pigmentosum variant and advanced cutaneous squamous-cell carcinoma. Acta Oncol. 2022, 61, 1140–1142. https://doi.org/10.1080/0284186X.2022.2109425.

77. Şahin, E.A.; Taşkıran, E.Z.; Pöş, K.; Aydın, B.; Utine, E. Recurrent squamous cell carcinoma and a novel mutation in a patient with xeroderma pigmentosum: A case report. J. Med. Case Rep. 2022, 16, 306. https://doi.org/10.1186/s13256-022-03524-2.

78. Scheer, V.; Schmalz, O.; Lehmann, P.; Hofmann, S.C.; Wesselmann, U. Three-year disease-free remission in a xeroderma pigmentosum patient after adjuvant anti-PD-1 therapy. Eur. J. Cancer 2022, 173, 207–209. https://doi.org/10.1016/j.ejca.2022.06.050.

79. Fernandez, E.R.; Tamura, D.; Khan, S.G.; Momen, S.; Fassihi, H.; Sarkany, R.; DiGiovanna, J.J.; Kraemer, K.H. Retrospective study of efficacy and adverse events of immune checkpoint inhibitors in 22 xeroderma pigmentosum patients with metastatic or unresectable cancers. Front. Oncol. 2023, 13, 1282823. https://doi.org/10.3389/fonc.2023.1282823.

80. Boziou, M.; Dionyssiou, D.; Dionyssopoulos, D.; Lazaridou, E.; Lallas, A.; Apalla, Z. Can Cemiplimab Become a Life-Changer in Xeroderma Pigmentosum? Dermatol. Pract. Concept. 2023, 13, e2023160. https://doi.org/10.5826/dpc.1303a160.

81. Zalaquett, N.G.; Kreidieh, L.; Youssef, B.; Mourad, M.; Kreidieh, F. Case report: Neoadjuvant-intent pembrolizumab resulted in complete response in a xeroderma pigmentosum patient with locally advanced resectable cutaneous squamous cell carcinoma of the nose. Front. Med. 2024, 11, 1488400. https://doi.org/10.3389/fmed.2024.1488400.

82. Gambichler, T.; Hyun, J.; Oellig, F.; Becker, J.C.; Kreuter, A. Immune checkpoint inhibitors for children with xeroderma pigmentosum and advanced cutaneous squamous cell carcinoma: A case presentation and brief review. J. Dtsch. Dermatol. Ges. 2025, 23, 303–308. https://doi.org/10.1111/ddg.15648.

83. Proietti, I.; Pirisino, R.; Azzella, G.; Coppolelli, V.; Greco, M.E.; Casciani, E.; Potenza, C.; Filippi, L. A Rare Case of Xeroderma Pigmentosum: Nivolumab Treatment for Three Cutaneous Malignancies with Clinical and Metabolic Imaging Correlation. Diagnostics 2025, 15, 979. https://doi.org/10.3390/diagnostics15080979.

84. Brufau-Cochs, M.; Gómez-Martínez, S.; Aya, F.; Serra-García, L.; Puig, S.; Malvehy, J.; Podlipnik, S. Pembrolizumab therapy leads to resolution of lentigines and tumour control in a patient with xeroderma pigmentosum and advanced cutaneous squamous cell carcinoma. Clin. Exp. Dermatol. 2026, 51, 1101–1103. https://doi.org/10.1093/ced/llag029.
